# Genetic Characterization and Linkage Disequilibrium Estimation of a Global Maize Collection Using SNP Markers

**DOI:** 10.1371/journal.pone.0008451

**Published:** 2009-12-24

**Authors:** Jianbing Yan, Trushar Shah, Marilyn L. Warburton, Edward S. Buckler, Michael D. McMullen, Jonathan Crouch

**Affiliations:** 1 Genetic Resources Program, International Maize and Wheat Improvement Center, Mexico City, Mexico; 2 Department of Bioinformatics, International Crops Research Institute for the Semi-Arid Tropics, Hyderabad, India; 3 National Maize Improvement Center of China, China Agriculture University, Beijing, China; 4 Corn Host Plant Resistance Research Unit, United States Department of Agriculture, Agricultural Research Service, Starkville, Mississippi, United States of America; 5 Institute for Genomic Diversity, Cornell University, Ithaca, New York, United States of America; 6 Plant, Soil and Nutrition Research Unit, United States Department of Agriculture, Agricultural Research Service, Ithaca, New York, United States of America; 7 Department of Plant Breeding and Genetics, Cornell University, Ithaca, New York, United States of America; 8 Division of Plant Sciences, University of Missouri, Columbia, Missouri, United States of America; 9 Plant Genetics Research Unit, United States Department of Agriculture, Agricultural Research Service, Columbia, Missouri, United States of America; University of Georgia, United States of America

## Abstract

A newly developed maize Illumina GoldenGate Assay with 1536 SNPs from 582 loci was used to genotype a highly diverse global maize collection of 632 inbred lines from temperate, tropical, and subtropical public breeding programs. A total of 1229 informative SNPs and 1749 haplotypes within 327 loci was used to estimate the genetic diversity, population structure, and familial relatedness. Population structure identified tropical and temperate subgroups, and complex familial relationships were identified within the global collection. Linkage disequilibrium (LD) was measured overall and within chromosomes, allelic frequency groups, subgroups related by geographic origin, and subgroups of different sample sizes. The LD decay distance differed among chromosomes and ranged between 1 to 10 kb. The LD distance increased with the increase of minor allelic frequency (MAF), and with smaller sample sizes, encouraging caution when using too few lines in a study. The LD decay distance was much higher in temperate than in tropical and subtropical lines, because tropical and subtropical lines are more diverse and contain more rare alleles than temperate lines. A core set of inbreds was defined based on haplotypes, and 60 lines capture 90% of the haplotype diversity of the entire panel. The defined core sets and the entire collection can be used widely for different research targets.

## Introduction

Globally, maize is one of the most important food, feed, and industrial crops. Continued improvement and cultivar release with new target traits will require the most precise manipulation possible of the estimated 59,000 genes in the maize genome [1]. Targeted plant breeding must find the right combination of alleles at these genes using new technology and the more traditional “art” of the plant breeder, a process facilitated in maize by the wide range of genetic diversity available in the species [2]. Over 47,000 accessions of maize exist in genebanks around the world, about 27,000 of which are stored at the International Maize and Wheat Improvement Center (CIMMYT) [3]. This includes inbred lines, improved populations, traditional farmer's populations (landraces) and wild relatives. The majority of accessions are landraces, and to date, much of this germplasm has not been extensively characterized, and most of the landraces have yet to have been utilized in modern plant breeding. It is estimated that less than 5% of the germplasm available in the species is used in commercial breeding programs in the world, and in the U.S. less than 1% [2]. Lack of characterization data for the germplasm stored in the genebanks seems to be one of the impediments to increased use.

Inbred lines selected from hybrids, populations or landraces are the fundamental resources for maize breeding and genetic research. Molecular markers such as restriction fragment length polymorphisms and simple sequence repeats (SSRs) or microsatellites were widely used to estimate the relationships among diverse lines. Marker-based relationships have been used in breeding programs to estimate the coefficient of pedigree and to establish heterotic groups and patterns for hybrid breeding [4]–[6]; identify complex population structure and relative kinship (information necessary for association mapping studies) [7]; and to identify core subsets of lines with the maximum diversity from a larger collection of analyzed lines, to reduce the number of lines for study or utilization. For example, the Generation Challenge Program has established reference core sets for 12 crop species using molecular markers [http://www.generationcp.org/subprogramme1.php].

A number of studies have been presented for marker based diversity investigation focusing on specific germplasm with limited sample sizes (generally less than 300 inbred lines), including U.S. Corn Belt lines [8]–[9], European temperate lines [10], Chinese temperate lines [11] , and tropical [4], [5] and subtropical [6], [12] lines. There are also a few studies focused on more diverse mixes of germplasm [13]–[15]. A typical study was presented by Liu et al., [15] who studied a well represented collection of 260 lines including 4 major known subgroups (stiff-stalk, non-stiff-stalk, tropical and subtropical, and “mixed”). These lines form a diversity association mapping panel used in a number of studies [7], [16]–[17]. Maize germplasm naturally forms two major groups, temperate and tropical (including subtropical) based on the environmental and day length characteristics of the planting areas in the world. These two groups formed over thousands of years after maize migrated out of its tropical center of origin in Mexico. This suggests that there is much more diversity in tropical lines, a suggestion well supported by past marker studies [13]–[16]. Many useful alleles for improving temperate maize may be hidden in the tropical germplasm and should be uncovered for continued future improvement. For example, a recent study identified a gene, *lycopene epsilon cyclase*, related with provitamin A content in the maize kernel. The diversity of alleles of this gene was investigated using an allele mining strategy and has demonstrated that there is a much higher frequency for alleles favorable to human health in tropical lines than in temperate lines [17].

Association mapping using diverse genotypes in plants is a new and powerful tool that has begun to yield promising results in identifying the functional variation in both known and unknown genes associated with important agronomic and economic traits [for a summary, see review 18]. The breakdown of linkage disequilibrium (LD) across the genome of an organism is a key factor affecting the precision and accuracy provided by association mapping, and is in turn affected by many genetic and non-genetic factors, including recombination, drift, selection, mating pattern, and admixture [19]–[21]. There are several statistical parameters to estimate the extent of LD [22], and *r^2^*, the squared value of the correlation coefficient of the allelic states of two given polymorphic loci, is the most commonly used. Levels of LD in maize have been reported in multiple studies. Tenaillon et al., [23] sequenced 21 loci located on chromosome 1 in 25 individuals including 16 exotic landraces and nine inbreds. They estimated that the LD decay distance was less than 1000 bp in landraces. Based on results from sequencing 6 genes in 102 diverse inbred lines, Remington et al., [24] found that rates of LD decay were highly variable with an average of less than 2000 bp. However, in commercial inbred lines, LD decay may be slower and linkage blocks may extend more than 100Kb based on the study of 18 maize genes in 36 maize inbreds [25]. For regions that have experienced strong selective sweeps, LD may extend over 500 kb or more [26]–[28]. However, all LD studies to date have been based on a limited number of loci and genotypes. It would be valuable to estimate maize LD decay at the whole genome level and with a larger, globally representative sample of maize genotypes.

Another class of marker, single nucleotide polymorphisms (SNPs) is present in all plant and animal genomes in huge numbers. Nearly one million maize SNPs are currently available in public databases [www.panzea.org]. Several high throughput genotyping platforms have been developed for commercial use [29], and provide opportunities for the maize community to speed up research progress for large scale diversity analysis, high density linkage map construction, high resolution quantitative trait locus (QTL) mapping, LD analysis and genome-wide association studies. A barley GoldenGate assay with 1524 SNPs was developed and used to estimate the diversity, LD, population structure and SNP-trait associations in a collection of diverse barley varieties [30]. Recently, Hamblin et al., [31] compared analyses based on 89 SSRs to analyses based on 847 SNPs in the same maize collection of 259 inbred lines. The resolution in measuring genetic distance using SNPs based on allele-sharing was lower than the more polymorphic SSRs. Yu et al [32] estimated that the power of 1000 SNPs was similar to 100 SSRs for estimating population structure and relative kinship. The possibilities to automate SNPs will allow a much higher number of them to be used cheaply in characterization studies, overcoming the lower genetic information imparted by each SNP. The ability to quickly estimate genetic structure in populations, and LD structure in genomes, will greatly speed the identification and utilization of new and useful alleles for plant improvement.

With the continuing efforts of over 40 years, CIMMYT has selected and released more than 500 inbred maize lines (most of which are tropical and subtropical). These were selected from pools and populations of highly variable germplasm, including landraces from all over Latin America, and some germplasm from temperate populations mixed in as well. Studies have already been performed to investigate the diversity of some of the selected inbred lines [4]–[6], [13] using SSR markers, but a larger study of CIMMYT maize inbred lines, compared to lines from other breeding programs would allow a better estimation of how public maize breeding lines are related to, and compliment, each other. In this study, we applied a custom 1536 SNP GoldenGate assay to genotype a collection of lines chosen to represent the global maize diversity available in public maize breeding programs. The collection includes 351 lines selected from a tropical association mapping panel (containing CIMMYT and other public program breeding lines) and 281 lines from a mostly temperate association mapping panel (well characterized in previous studies [15]–[17]). Twenty one CML lines with the same name are included in both panels. The CMLs were developed at CIMMYT, so the original genotypes went into the first panel listed above, and these lines were brought by Prof. M. Goodman to North Carolina State University in the early 1980s and have been maintained in the USA since their introduction. This was the source of the CMLs in the second panel listed above. The objectives of this study were to: 1) estimate the diversity within a global maize collection; 2) compare the diversity between temperate and tropical germplasm; 3) analyze the population structure and relative kinship; 4) investigate linkage disequilibrium and how breakdown relates to chromosomes, minor allelic frequency (MAF), sample size and subgroup of different geographic origins; 5) determine how many lines must be included in a core set to capture at least 90% of the allelic diversity present in the entire collection; and 6) verification of genetic identity of different seed sources with the same name using SNP markers.

## Results

### SNP Performance and Quality

Of the 1536 maize SNPs present in our oligo pool assay (OPA), 1311 SNPs (85%) were successfully called in the 632 lines with less than 20% missing data. Eighty two of the 1311 successful assays were monomorphic in all 632 lines, which may have been caused due to errors in sequencing and/or SNP development. A final total of 1229 SNPs were used for further data analysis. Heterozygosity ranged from 0 to 9.9%, with an average of 2.5%, well within expected ranges for residual heterozygosity found in inbred maize lines.

One sample (CML312) was repeated in four different plates as a control. Over 98% of the data points were identically scored in the four repeated samples. Only one type of genotyping error was found, where a SNP was called as homozygous in one plate but heterozygous in the repeated sample in another plate. For any given pair of repeated samples, the genotyping errors ranged from 0.08% to 1.7% with an average 0.8%; this is comparable with other maize SNP assays [33].

### Summary of SNPs and SNP Haplotypes

The 1229 SNPs were mapped *in silico* onto the maize genome and the detailed map and information is available at http://cmap.cimmyt.org/cgi-bin/cmap/viewer?data_source=CMAPsaved_link_id=5. All SNPs were well distributed across the 10 chromosomes, and coverage ranged from 65 SNPs on chromosome 6 to 211 SNPs on chromosome 1. This represented 538 loci with an average 2.3 SNPs per locus (Table 1). Among the 538 loci, 211 contained only one SNP, and the other 327 contained 2 or more SNPs with an average of 3.1 SNPs per locus (Table 1). To extract the most useful information from the SNP data (by creating multiple alleles and thus increasing the genetic information), haplotypes were constructed for those loci with more than one SNP. The 327 SNP haplotypes had a total of 1749 alleles (Table 2). They ranged from 2 to 41 alleles per locus, with the number of alleles generally increasing with the number of SNPs scored within each locus (Table 3).

**Table 1:** Summary of SNPs used in this study.

Chr.	SNP Number	Unique loci		Minor Allelic Frequency		
		2+SNPs	1SNP	≥0.05	≥0.1	≥0.2
1	211	61	19	178	148	98
2	166	37	33	124	93	60
3	131	33	30	92	77	52
4	132	36	26	101	75	45
5	128	36	30	102	79	43
6	65	18	14	50	38	25
7	112	22	17	90	75	47
8	102	26	13	82	75	48
9	83	21	13	63	56	38
10	73	16	13	61	45	34
Unknown	26	21	3			
Total	1229	327	211	943	761	490

**Table 2:** Properties of SNPs and SNP haplotypes.

Marker	Loci	Represent unique locus*	Alleles
SNP	211	211	422
	1018	327	2036
SNP haplotype	327	327	1749

*SNPs from same locus within 10Kb region were combined and identified as a unique locus.

**Table 3:** Number of haplotypes (alleles) observed in each locus from which one or more SNPs were amplified.

SNPs	Locus	Allele number																			
		2	3	4	5	6	7	8	9	10	11	12	13	14	15	16	19	20	23	33	41
1	211	211																			
2	154	5	90	59																	
3	81		4	32	13	17	10	5													
4	52	1	3	3	14	10	9	3	1	4	1	1		1	1						
5	14				2	2	2	1	2	1	2	1		1							
6	13				2	2	1	2	1		1				2	2					
7	5							1	1				1				1	1			
8	4					1		1								1		1			
9	1											1									
10	1																				1
12	1																			1	
13	1																		1		
Total	538	217	97	94	31	32	22	13	5	5	4	3	1	2	3	3	1	2	1	1	1

### Allelic Frequency of SNPs and SNP Haplotypes

**Figure 1:**
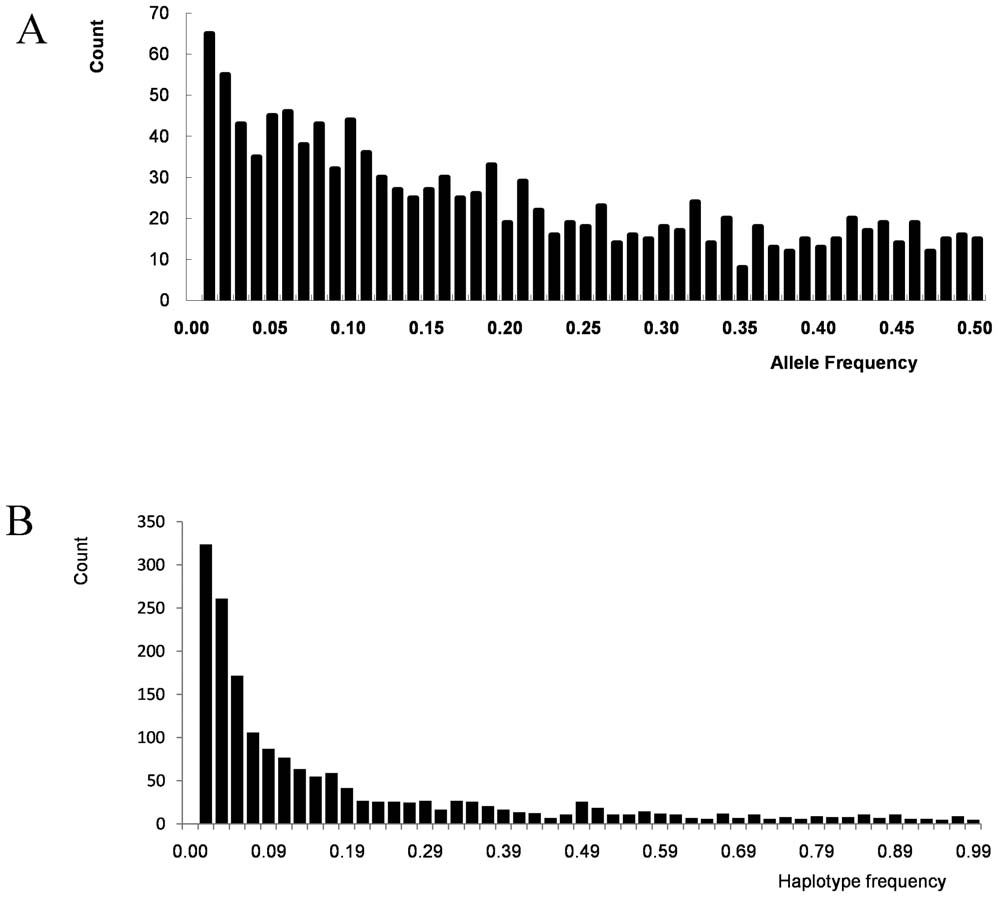
Allele frequency for total SNPs. SNPs scored as biallelic markers are in the top panel, and scored as SNP haplotypes are in the bottom panel.

The single SNPs are bi-allelic, with a continuous allele frequency distribution (Figure 1a). The SNP haplotypes have a large number of alleles, but most are rare in the population; over half have an allelic frequency less than 0.1 (Figure 1b). The proportion of total SNPs that were polymorphic between pairs of different lines (polymorphism ratio) ranged from 0.1 to 47.4% with an average value of 26.2%. The highest level of polymorphism occurred between the lines CML186 and MEF15-55-2, and the lowest occurred between NC364 and NC362 which were two related lines. The average polymorphic ratio for any given line to the other 631 lines ranged from 19.3% for CML35 to 41.1% for CML186. Because all SNPs used in this study were developed from sequencing the set of 27 lines that were used to develop the nested association mapping (NAM) population [34], there may be some ascertainment bias that may affect the frequencies of the alleles in further SNP-based studies [31].

### Population Structure and Relative Kinship

**Figure 2:**
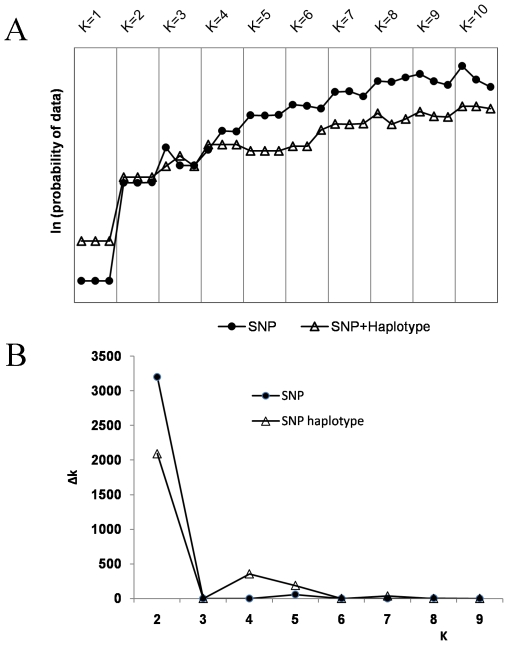
Estimated ln(probability of the data). Ln(probability of the data) was calculated for K ranging from 1 to 10 (top panel) and delta K values for SNPs and SNP haplotypes for K from 2 to 9 (bottom panel).

In past studies, the population structure of the 281 lines from the Cornell association mapping panel has been ascertained using SSR and SNP markers [16], [31], and determined to consist of four clusters referred to as stiff-stalk (SS), non-stiff-stalk (NSS), tropical and subtropical (TS) and “mixed” subpopulations. The results based on SNPs or SNP haplotypes were consistent with those using only SSR markers [31], suggesting that bi-allelic SNP markers can also be used for population structure characterization. We ran STUCTURE for K (number of fixed subgroups or clusters) ranging from 1 to 10 on the entire set of inbred lines using all SNPs scored as biallelic markers, and then using individual SNP plus SNP haplotype data which combined linked SNPs into haplotypes. The likelihood value of this analysis is shown in Figure 2. Likelihood increases continuously and no obvious inflection point was observed either for SNP or SNP haplotypes. This could imply that the lines included in the analysis were very diverse as well as highly mixed.

**Figure 3:**
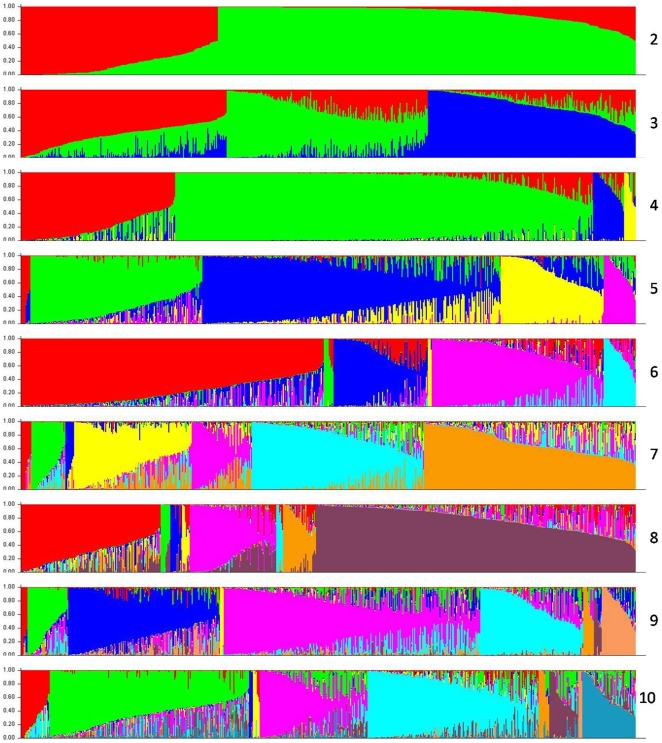
Estimated population structure of the diverse inbred maize lines in the study. Each of the 632 individuals is represented by a thin vertical line, which is partitioned into k colored segments that represent the individual estimated membership to the k clusters.

However, the most significant change was observed increased when K was increased from one to two, and based on the origin, pedigree, and breeding history of germplasm in this study, we know that we may divide the lines between temperate and tropical/subtropical subgroups. Structure results of K = 2 was the best possible partition as they showed a high consistency with known pedigree history and geographic origin, and significant delta K values (Figure 2). Thus, 156 lines, mostly from the NSS and SS subgroups [16] were assigned to the temperate subgroup, and 365 lines, including most of the lines selected by CIMMYT and the TS subgroup from the results of Flint-Garcia et al [16] were assigned to the tropical/subtropical subgroup. In addition, another 111 lines were assigned to “mixed” subgroup (Table S1). A further study of the partitioning of lines can be seen in Figure 3, which is the Structure graphical representation of the placement of each line in the study into its corresponding cluster, for K ranging from 2–10. Such a graph shows the number of lines in each cluster, and the percent mixing of each line within each cluster, a useful visualization of admixture.

**Figure 4:**
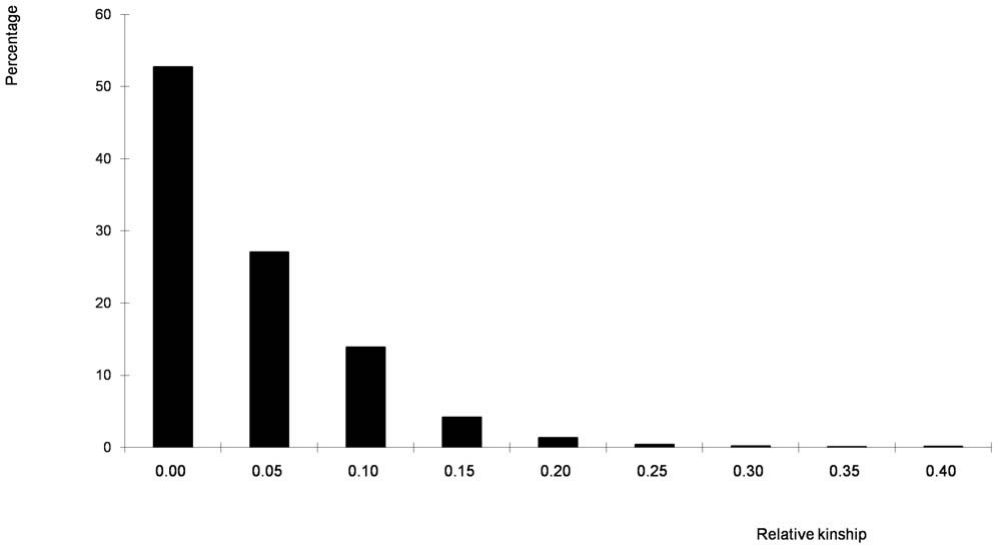
Distribution of pairwise relative kinship values. Values equal to or greater than 0.40 were gouped as 0.40.

Molecular markers can be used to calculate relative kinship between pairs of individuals in a study, which provides useful information for quantitative inheritance studies [7]. The relative kinship reflects the approximate identity between two given individuals over the average probability of identity between two random individuals [7]. In this study, 700 informative SNPs with MAF>0.1 and little or no missing data were used to estimate the relative kinship in the set of 632 lines. As shown in Figure 4, about 50% of the pairwise kinship estimates were close to 0, indicating that the lines were unrelated. The remaining estimates ranged from 0. 05 to 1, with a continuously decreasing number of pairs falling in higher estimate categories. The kinship analysis indicates complex familial relationships among the 632 lines, matching with the known pedigree history.

### Diversity Comparison between Tropical and Temperate Subgroups

To compare diversity in the tropical and subtropical subgroup with the diversity in the temperate subgroup, equal numbers of genotypes were selected randomly from both germplasm pools and analyzed. As shown in Table 4, the tropical pool has captured more SNP alleles and haplotype diversity than the same sized temperate pool sample. For example, 40 genotypes capture 79.2 and 71.8% of the total reported haplotype alleles from the tropical/subtropical pool and the temperate pool, respectively. Approximately 25% of the total haplotypes are not found in the temperate pool, and only 8% of the total haplotypes are not found in the tropical/subtropical pool. Of the 1229 SNPs, 49 are found only in one subgroup (36 from tropical or subtropical, 6 from temperate, and 7 from mixed). Of the 1749 haplotypes, 286 are found only in one subgroup (191 from tropical and subtropical, 45 from temperate and 50 from mixed); and 131 haploytpe alleles are found in only one inbred line, about half of which (64) are from tropical and subtropical genotypes.

**Table 4:** Diversity comparison of tropical (A) and temperate (B) and subgroups.

Subset size	Allele (number)				within-group diversity (%)*				total diversity (%)#			
	SNP		Haplotype		SNP		Haplotype		SNP		Haplotype	
	A	B	A	B	A	B	A	B	A	B	A	B
10	2155	2162	1015	999	89.2	92.8	61.7	74.6	87.7	88.0	56.9	56.0
20	2309	2268	1236	1162	95.5	97.4	75.1	86.7	93.9	92.3	69.2	65.1
40	2390	2322	1414	1281	98.9	99.7	85.9	95.6	97.2	94.5	79.2	71.8
60	2414	2329	1503	1323	99.9	100.0	91.3	98.7	98.2	94.8	84.2	74.1
80	2417	2329	1559	1340	100.0	100.0	94.7	100.0	98.3	94.8	87.3	75.1
100	2417	2329	1590	1340	100.0	100.0	96.6	100.0	98.3	94.8	89.1	75.1
120	2417	2329	1613	1340	100.0	100.0	98.0	100.0	98.3	94.8	90.4	75.1
140	2417	2329	1632	1340	100.0	100.0	99.1	100.0	98.3	94.8	91.4	75.1
156	2417	2329	1643	1340	100.0	100.0	99.8	100.0	98.3	94.8	92.0	75.1
365	2417		1646		100.0		100.0		98.3		92.2	

*the ratio of captured alleles within the subgroup compared to a given subset.

# the ratio of captured alleles in the whole panel compared to a given subset.

### Core Sets

A core set of lines from the population of 632 lines studied here was created that captures the maximum diversity of the SNP haplotypes. As shown in Table 5, 20 lines can capture 74.7% of the 1749 haplotypes found in the 632 lines; and 40, 60, 80 and 100 lines can capture 85.0, 90.1, 93.0 and 95.1% of the haplotypes, respectively. To recover all the 1749 haplotypes, 212 lines were needed.

**Table 5:** Alleles captured by subsets of the data for SNP haplotypes.

Subset size	Haplotypes obtained	Captured (%)	Inbreds†
20	1333	74.7	H84, IDS91, K4, Ki44, L109, L128, L172, L292, L419, Mo45, NC236, NC264, NC302, NC306, NC342, NC362, Tzi16, Va26, Va99, Wf9
27*	1332	74.6	B73, B97, CML103, CML228, CML247, CML277, CML322, CML333, CML52, CML69, HP301, Il14H, Ki11, Ki3, Ky21, M162W, M37W, Mo17, Mo18W, MS71, NC350, NC358, Oh43, OH7B, P39, Tx303, Tzi8
40	1517	85.0	B73Htrhm, CI66, CM174, CML258, CO106, H84, IDS69, IDS91, K4, K55, Ki44, KY226, L108, L12, L128, L165, L18, L202, L268, L284, L292, L337, L363, L374, L398, L414, L67, L91, MoG, NC260, NC264, NC300, NC302, NC342, NC362, SC213R, Tzi16, Va17, Va35, Va99
60	1608	90.1	B115, B75, CML258, CML312, CML38, CO106, F2834T, H84, I137TN, I205, IDS91, K4, K55, Ki44, KY226, L101, L108, L109, L111, L114, L12, L128, L131, L18, L192, L198, L209, L217, L245, L256, L264, L284, L291, L292, L30, L349, L36, L374, L39, L419, L64, L67, L7, L91, Mo46, MoG, NC236, NC260, NC264, NC296, NC300, NC302, NC338, NC342, NC362, Oh40B, Tzi16, VA102, Va35, Va99
80	1660	93.0	38-11, A272, A6, A661, B115, B75, CML14, CML258, CML261, CML312, CO125, F2834T, H84, I137TN, I205, IDS91, K148, K4, K55, Ki44, KY228, L108, L109, L111, L114, L119, L12, L128, L154, L170, L18, L19, L192, L198, L200, L208, L245, L248, L258, L284, L290, L292, L296, L30, L333, L349, L368, L373, L417, L419, L436, L437, L453, L454, L54, L59, L64, L67, L79, L91, L99, Mo17, NC236, NC250, NC260, NC264, NC296, NC300, NC302, NC338, NC342, NC362, Oh40B, Sg18, T232, VA102, Va17, Va35, Va99, W182B
100	1698	95.1	A272, A441-5, A680, B115, B73Htrhm, C49A, CI66, CM37, CML14, CML258, CML261, CML312, CML341, I137TN, I205, IDS91, K4, K55, Ki11, Ki44, KY228, L108, L109, L114, L119, L12, L128, L131, L154, L160, L170, L173, L18, L181, L185, L189, L200, L201, L209, L230, L232, L245, L246, L248, L250, L262, L27, L284, L291, L293, L296, L297, L309, L317, L328, L333, L334, L343, L349, L36, L363, L368, L373, L39, L406, L414, L419, L437, L438, L445, L454, L5, L51, L54, L578, L59, L64, L67, L71, L83, L91, L99, Mo47, MoG, NC236, NC264, NC294, NC296, NC300, NC302, NC338, NC342, NC362, Oh40B, SA24, Tzi16, VA102, Va26, Va35, Va99

*Represent the 27 parents of the NAM population.

† The detailed pedigree information of the inbreds, including group classification by Structure, can be found in Table S1.

### Verification of Genetic Identity of Different Seed Sources

Twenty one CMLs included in both panels were used to estimate how large the differences can grow between lines with the same name that have been maintained separately for over 30 years. Differences measured by SNP markers may be attributed to drift or selection on residual heterozygosity in the lines before they were separated; gene flow due to seed or pollen mixing; or labeling mistakes. For the “same” line, the ratio of mismatched SNP markers varied between 0.2% and 19.5%, with an average 4.1% (Table 6). The ratio of mismatch is more than 10% for 4 of the 21 lines and two lines (CML322 and CML328) reached 20% mismatch.

**Table 6:** Comparison of the 21 “CML” lines with the same name stored at CIMMYT and North Carolina State University, respectively.

Lines	Different SNPs	Total SNPs	difference (%)
CML103	65	1182	5.5
CML108	20	1177	1.7
CML218	9	1160	0.8
CML220	100	999	10.0
CML228	56	1148	4.9
CML238	29	1134	2.6
CML254	3	1182	0.3
CML261	8	1199	0.7
CML281	16	1197	1.3
CML287	3	1204	0.2
CML321	6	1212	0.5
CML322	231	1182	19.5
CML323	2	1175	0.2
CML328	171	867	19.7
CML331	10	1216	0.8
CML333	5	1197	0.4
CML341	34	1193	2.8
CML38	39	1085	3.6
CML69	27	1140	2.4
CML91	150	1109	13.5
CML92	18	1206	1.5
Average	47.7	1150.7	4.1

### Linkage Disequilibrium

There have been many LD analyses in maize [for a summary, see review 19], but most have been based on a limited number of individuals and loci. A truly global look at LD breakdown has been lacking to date. In this study, 1229 SNPs representing 538 loci were used to score a large diversity panel of 632 lines (both temperate and tropical), providing an opportunity to investigate LD at the whole genome level, as well as to study the effect of the following factors on LD: chromosomes, MAF, sample size, and subgroups of different geographic origins.

#### Chromosome and genetic distance

**Figure 5:**
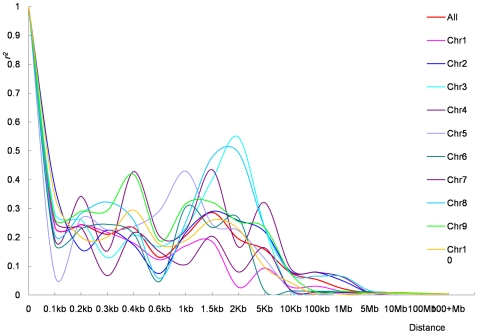
Linkage disequillibrium across the maize 10 chromosomes measured with 914 SNPs. Only SNPs with a minor allele frequency greater than 0.05 are shown.

All 943 SNPs with MAF≥0.05 that were mapped *in silico* to the maize physical map were used for this analysis (Table 1). The mean *r^2^* pooled over all ten chromosomes in different categories of map distance are summarized in Table 7. The distributions of *r^2^* with respect to the physical distance for each chromosome as well as all chromosomes are presented in Figure 5 and Table 8. A rapid decline was observed with increasing physical distance, also seen in previous studies [19]. Linkage disequilibrium decay varies over different chromosomes with 1.5–2 kb in chromosome 1, 2–5 kb in chromosomes 6 and 10 and 5–10 kb in the remaining 7 chromosomes, with an average of 5–10 kb. Average LD decay is a little greater to one of the previous estimates reported [24], but variation between chromosomes implies that LD decay estimation only based on a single chromosome or a limited number of loci may be biased. Figure 5 also shows that mean *r^2^* between 0 and 2kb did not decrease in a continuous fashion with the increase in physical distance. Even for distances of less than 100bp, mean *r^2^* was only 0.237 (Table 7).

**Table 7:** Mean LD among all SNPs with a minimum allelic frequency greater than 0.05, over different map distances and across 10 chromosomes.

Distance	N	Mean (*r^2^*)	SD (*r^2^*)	25^th^ percentile *r^2^*	50^th^ percentile *r^2^*	75^th^ percentile *r^2^*	Minimum number SNP required to cover genome
0–0.1kb	114	0.237	0.283	0.019	0.121	0.342	>24,000,000
0.1–0.2kb	167	0.244	0.278	0.044	0.128	0.33	24,000,000-12,000,000
0.2–0.3kb	121	0.211	0.27	0.029	0.099	0.31	12,000,000-8,000,000
0.3–0.4kb	98	0.234	0.28	0.022	0.099	0.376	8,000,000-6,000,000
0.4–0.6kb	80	0.131	0.212	0.013	0.055	0.159	6,000,000-4,000,000
0.6–1kb	122	0.207	0.278	0.013	0.096	0.283	4,000,000-2,400,000
1–1.5kb	100	0.287	0.332	0.032	0.126	0.408	2,400,000-1,600,000
1.5–2kb	53	0.213	0.304	0.017	0.032	0.25	1,600,000-1,200,000
2–5kb	131	0.158	0.255	0.013	0.044	0.168	1,200,000-480,000
5–10kb	34	0.077	0.126	0.011	0.033	0.091	480,000-240,000
10–100kb	68	0.053	0.139	0.003	0.012	0.032	240,000-24,000
0.1–1Mb	479	0.022	0.099	0.001	0.004	0.012	24,000-2,400
1–5Mb	2285	0.008	0.014	0.001	0.003	0.009	2,400-1,600
5–10Mb	2911	0.006	0.011	0.001	0.003	0.007	1,600-240
10–100Mb	23701	0.005	0.011	0	0.002	0.006	240-24
>100Mb	19618	0.004	0.007	0	0.002	0.005	NA

**Table 8:** Average LD decay distance of the 10 chromosomes for *r^2^* greater than 0.1.

Chr.	LD decay (kb)
1	1.5–2
2	5–10
3	5–10
4	5–10
5	5–10
6	2–5
7	5–10
8	5–10
9	5–10
10	2–5
Average	5–10

#### LD and MAF

**Figure 6:**
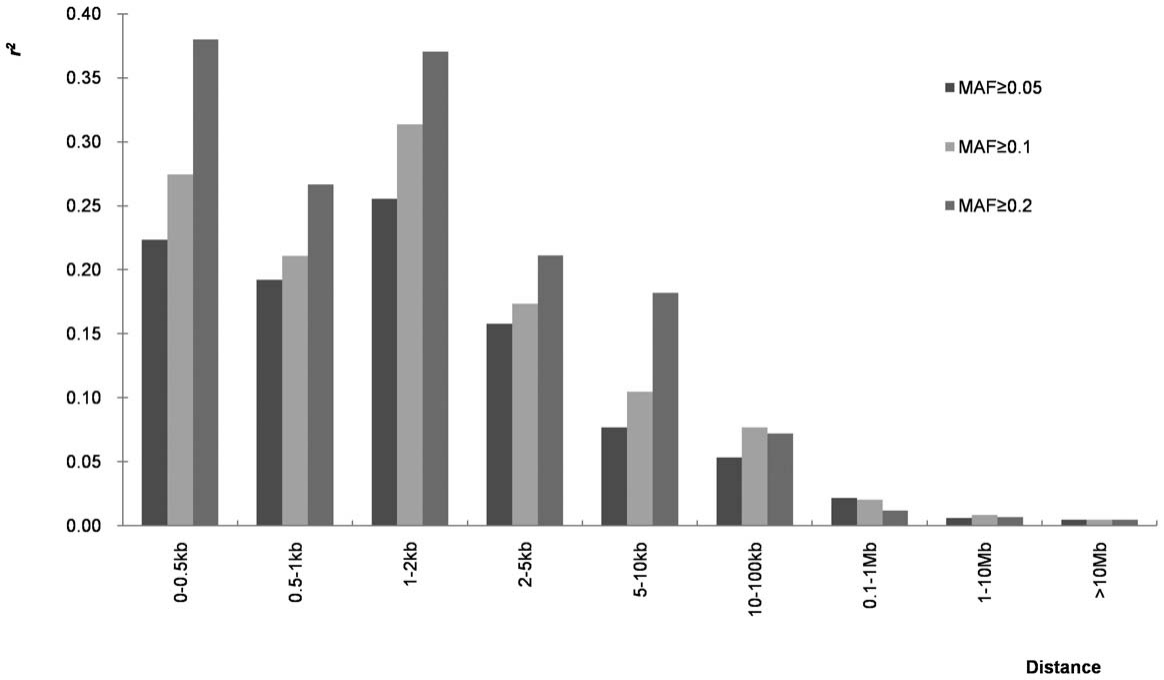
Mean LD estimates at different physical distances for three different minimum threshold cutoff levels for minimum allele frequency. Mean LD estimates are pooled over all chromosomes, and three different minimum threshold cutoff levels for minimum allele frequency are shown.

Three different minimum allelic frequency thresholds (0.05, 0.1 and 0.2) were used to study the effects of MAF on the extent of LD. As can be seen in Figure 6, MAF significantly affects mean *r^2^*, especially for short distances (between 0 and 10 kb). Mean *r^2^* increased significantly with MAF. For example, from 0–0.5 kb, mean *r^2^* for MAF≥0.05 was 0.22, but increased to 0.27 and 0.38 using data with MAF≥0.1 and 0.2, respectively.

#### LD and sample size

**Figure 7:**
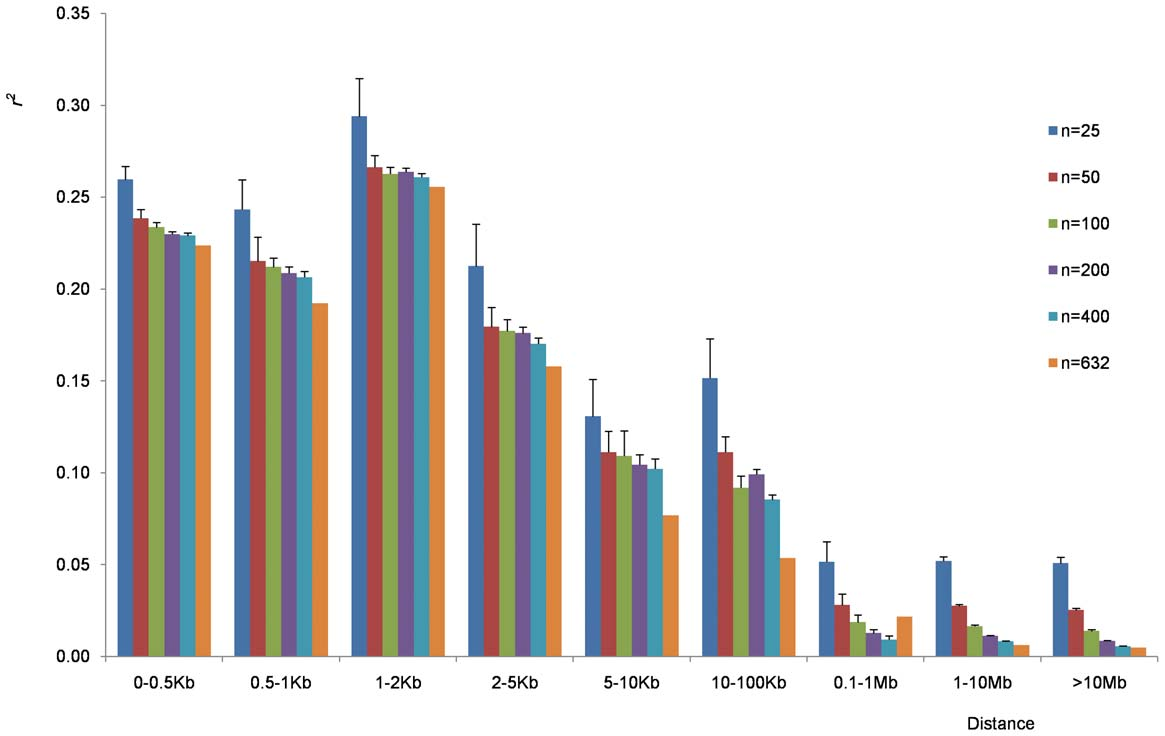
Mean LD estimates for different physical distances for six different sample sizes. Mean LD estimates are pooled over all chromosomes, and six different sample sizes using SNPs with minor allele frequency greater than 0.05 are shown.

Five subsets of different sample sizes (n = 25, 50, 100, 200, and 400) were randomly selected from the entire set with ten repetitions each using SNPs with MAF≥0.05 to study the effect of sample size on the extent of LD. As shown in Table 9 and Figure 7, LD estimates are greater when sample size is smaller, and this trend is more noticeable for LD measured across marker interval greater than 5kb. Few significant differences for LD estimates were found with sample sizes greater than 50 and marker distances less than 10 kb. More significant effects on the estimation of LD due to sample size are observed for long range LD estimates (marker intervals greater than 10 kb).

**Table 9:** Correlations between pairwise estimates of LD obtained from different sample sizes compared to the entire sample of 632 lines.

Entire sample	Selected sample	Correlations of estimates between selected and entire sample	
		*r^2^*	MAF
632	25	0.55 (0.51–0.57)	0.79 (0.77–0.81)
632	50	0.75 (0.73–0.76)	0.89 (0.88–0.91)
632	100	0.79 (0.78–0.80)	0.95 (0.94–0.95)
632	200	0.89 (0.89–0.89)	0.98 (0.98–0.98)
632	400	0.91 (0.90–0.91)	0.99 (0.99–0.99)

Each subsample was taken 10 times to obtain a mean and range.

Correlations of *r^2^* estimates between the randomly selected subsets and the entire set increase with increasing subsample size. Correlations between ten selected samples of n = 25 and the entire sample ranged from 0.51 to 0.57 with an average of 0.55. When sample size was increased to 50, the average correlation value increased to 0.75. Correlations reach 0.91 when the selected sample size is increased to 400 (Table 6). Similar patterns are also seen for the correlation of MAF between the subsets and the entire population, and correlations increased from 0.79 to 0.99 when selected sample size increased from 25 to 400. As seen in the previous section, as MAF increases, LD also increases for low MAF, so a small sample size may lead to an incorrect measurement of LD directly or indirectly by leading first to an incorrectly low MAF.

#### LD and subgroups of different geographic origins

**Figure 8:**
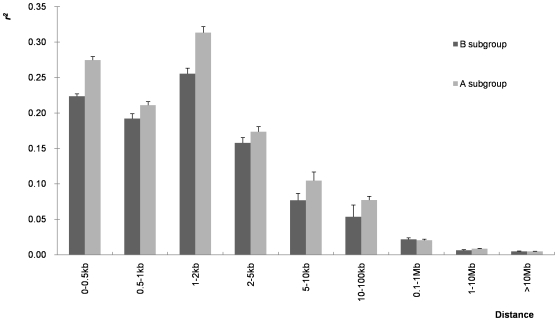
Mean LD estimates at different physical distances for tropical and temperate subgroups. Mean LD estimates are pooled over all chromosomes, and two subgroups (A = tropical, B = temperate) at sample size = 80 are shown, using SNPs with a minor allele frequency of 0.05.

Eighty genotypes per subgroup, which together capture over 90% of the SNP diversity within each subgroup, were randomly selected from the entire set with ten repetitions each using SNPs with MAF≥0.05 to study the effect of subgroups of different geographic origins on the extent of LD. LD across marker intervals in the temperate subgroup is much greater than in the tropical/subtropical subgroup, and LD across marker intervals within either subgroup is greater than in the random sample selected from the entire panel (Figures 7 and 8).

## Discussion

### Core Sets

Defining a core subset that captures the maximum diversity from a bigger collection is a useful tool for germplasm characterization, breeding and genetic research. The number of inbred lines that can capture the maximum number of alleles generally defines the most useful size for a core subset of the entire population [15]. One hundred and two hundred and twelve lines can capture 95% and 100% of the 1749 haploytype alleles found in the 632 lines in this study. These subsets can be used as two possible core sets for future research targets such as allele mining and association mapping. Different core subsets of 100 lines chosen at random should also capture approximately 95% of the diversity of the entire panel, and different cores may be chosen for different research purposes. For example, considering that the 632 lines are very diverse and may not be adapted to any single environment, a new subset from this panel can be chosen with good adaption to a specific environment. Conversely, lines that may display good phenotypic expression of a desired trait may be chosen. It is important to keep in mind, however, that core subsets chosen with a specific purpose in mind are not random, so some of the allelic variation from the entire set of 632 lines may be missed.

### Genetic Diversity of Tropical and Temperate Germplasm Pool

To compare the diversity of the tropical and subtropical subgroup with the temperate subgroup, equal numbers of samples were selected randomly from both germplasm pools. The results are shown in Table 8. The genetic diversity in the tropical germplasm pool is much higher than that in the temperate pool, in agreement with a previous study using SSR markers [15]. Tropical and subtropical lines were found to contain many rare alleles in past studies [15] and are an important resource to find new functional alleles of desired traits and can be used broaden the genetic base of maize breeding populations or to find sequence variation for targeted introgression into temperate breeding lines in the future. In most breeding programs, few crosses are made between temperate and tropical lines due to adaptation issues for temperate lines in tropical regions and vice versa. However, the large number of rare and group specific alleles identified in the tropical and subtropical germplasm pool suggests that we consider the issue again. With the rapid development of molecular marker techniques in the past two decades, it is now reasonable to introduce exotic chromosomal segments into targeted materials without additional linkage drag, and will provide new resources for maize breeding, as suggested by Bernardo [35].

### Verification of Genetic Identity of Different Seed Sources

CIMMYT is one of the most important centers for maize germplasm collection, conservation and utilization, particularly in the developing world. More than 500 CIMMYT derived inbred maize lines (CMLs), have been released and used extensively to develop new hybrid maize varieties. In this study, we compared the genetic identity of 21 CML lines with same name but maintained in different labs for more than 30 years. Although 17 of the lines were still genetically similar, we found 4 lines with a mismatch ratio more than 10%, two of which reached nearly 20% mismatch. Genetically, this is a very low level of similarity for supposedly identical lines, suggesting that many heterozygous loci existed in the maize inbreds developed by CIMMYT which might have become fixed differently in different institutes; or that outcrossing has occurred at one or both institutions during seed increase and regeneration. This serves as a reminder for much care to be taken for future germplasm exchange and conservation, especially for genetic research.

### Structure of LD in Maize

In this study, we genotyped a large and diverse collection of 632 lines with 1229 SNPs from 538 loci to determine the global structure of LD in the maize genome. Fine scale coverage was only 2.3 SNP/gene on average, but this was enough to give us a rough picture of the structure of LD in maize at the whole genome level. Our results demonstrate that LD decline is variable across the chromosomes and not continual within a chromosome. More markers may have smoothed some of the discontinuity within a chromosome, but it also reflects the known complex genome structure of maize. Minimum allelic frequency is another factor that affects estimation of the extent of LD. Within the global decay distance of maize LD (5–10 kb), mean *r^2^* increases with the increase of MAF, and a similar phenomenon was also observed in other species [36]. Khatkar et al., [36] proposed that SNP pairs with similar allelic frequencies may increase estimates of *r^2^*. In this study, removing SNPs with very low MAFs also lead to lower numbers of SNPs available for study, which can also lead to bias of LD estimates. A small sample size (e.g. n = 25) can also lead to the biased estimation for LD. However, there are no significant differences for the mean *r^2^* when sample sizes are over 50, especially when the given extent interval of LD is less than 2 kb (Figure 7). However, a recent study in cattle demonstrated that a sample of 400 or more was required for reliable estimation if using D′ to measure LD [36]. Decay of LD is also greatly affected by the sequence diversity present in the samples used. The LD decay is more rapid in tropical and subtropical lines than in temperate lines when sample numbers are equivalent (Figure 8), because there is more sequence diversity in tropical and subtropical than temperate lines (Table 8).

### Genomewide Association Studies

Genome-wide association is a powerful tool that is widely used in human genetic studies [37] and is now being used in plants such as Arabidopsis [38] and more recently, maize [39]. Construction of a representative and genetically diverse panel of fixed lines is the first step for any successful association mapping study in plants. The population of lines studied here would be an ideal panel for maize association studies, because it is larger than any previously reported association mapping panel. For any panel of lines chosen for association mapping, controlling population structure is the key factor for improving statistical power and decreasing the false positive rate in gene discovery [7]. The present collection has some population substructure beyond the well understood partitioning of temperate and tropical germplasm, and the familial relatedness has been described and can thus be taken into account during association studies based on the present marker study.

Average *r^2^* can be used predict the power for genome-wide association studies that given numbers of markers will have. The average decline of LD distance for single marker association mapping in this panel is 5–10 kb (Table 4), which suggests 240,000 to 480,000 markers will be needed for whole genome scanning in maize, as the maize genome is known to extend over 2,400 Mb. Fortunately, however, considering that eighty percent of the maize genome consists of repetitive sequence, the actual number of SNPs required for genome-wide association studies can be considerably reduced if we develop SNPs specifically from expressed regions of the genome. However, further demonstrating the difficulties that will be faced in maize genome-wide studies, at an LD distance of 5–10 kb, the mean *r^2^* is only 0.077 (median *r^2^* = 0.033), implying that statistical power may be even lower for detecting the nucleotide changes encoding quantitative traits based on weak correlations between adjacent SNPs. Increasing marker density ten times to 4,800,000 may not increase power significantly since the mean *r^2^* only increases to 0.207 (median *r^2^* = 0.096). In addition, considerable variation exists for *r^2^* within a given LD distance. For example, the 50^th^ percentile for *r^2^* is very small (for almost all LD distances considered in this study (Table 4)), and less than half of the SNP pairs have an *r^2^* value greater than 0.15 in any measured LD distance. This variation may be caused by different LD in different chromosomal regions, implying that more markers might be needed overall for successful implementation of genome-wide association than the number predicted based on mean *r^2^*.

A previous simulation study showed that more power was achieved by increasing the number of individuals in the population than by increasing the SNP density within a candidate gene [40]. The simulations show that it is possible to detect a QTL/gene that accounts for as little as 5% of the total phenotypic variation for a trait when 500 individuals were genotyped with 20 SNPs within the candidate gene region (which corresponds to using 1 million SNPs to cover the entire genome) [40]. Recently, genome-wide association studies were performed to identify genes affecting height of adult humans in three studies [41]–[43] with large sample sizes (14,000–34,000) and more than one million SNPs. In total, 54 variants were identified that each explain 0.3–0.5% of the phenotypic variation. Other simulation results found only a 50% level of power to detect associated variants with a 0.5% effect on the phenotypic variation based on 5000 individuals in genome-wide association studies [44]. Based on the simulation and human studies mentioned above, the panel of individuals characterized in the present study should provide the power to detect nucleotide variants affecting quantitative traits in maize explaining at least 3–5% of the total phenotypic variation for candidate gene or genome wide association studies with about 20 SNPs per gene. This may be the minimum that would make marker assisted selection studies worthwhile for the identified genes.

### Conclusions

In this study, we used 632 diverse lines and 1229 SNPs derived from 538 loci to estimate the LD of maize in the whole genome level. The LD decay distance differed among chromosomes and ranged between 1 to 10 kb, increased with the increase of MAF and with smaller sample sizes, was much higher in temperate than in tropical and subtropical lines. These results provide useful information for understanding the maize genome structure and further genome-wide association studies.

## Materials and Methods

### Plant Material

Two independent panels of diverse/commercial inbred lines were included: one from Cornell University containing 281 temperate (and some tropical) maize inbreds [16]; the other panel was developed by CIMMYT and includes 351 mainly tropical and subtropical lines. Both panels have been used for association mapping, and both were chosen from a much larger collection of lines based on allele diversity using SSR markers. Between the two panels, therefore, most of the genetic variation of the world's public breeding programs is expected to be represented. Twenty one CML lines with the same name but different seed sources were included in both panels. The lines are listed in Table S1.

### SNP Discovery and Assay Development

All SNPs were developed from the coding regions of 582 candidate genes, about half of which comprised putative drought-related loci. To identify SNPs from the genomic sequences, primers were designed based on the sequences of selected loci and used to amplify products of 600–1000 bp in four test lines. Primers that amplified successfully were used to amplify the same genes in the 27 diverse inbred lines used as parents of the Nested Association Mapping (NAM) population [34]. Sequence data were aligned using Biolign software version 4.0.6 [45] and SNPs were extracted using TASSEL 2.0 [46]. More than 10,000 SNPs were discovered. The best SNPs for this assay were chosen based on the quality scores assigned by the Illumina Company, who developed the assay. Finally, an Illumina oligo pool assay (OPA) with 1536 good SNPs was developed from 732 amplified products representing 582 unique loci. A detailed list of the 1536 SNPs can be downloaded from (http://www.panzea.org/db/gateway?file_id=2007_candidate_snp).

### SNP Genotyping and Analysis

The SNP genotyping was performed on an Illumina BeadStation 500 G (Illumina, San Diego, CA) at the Cornell University Life Sciences Core Laboratory according to the manufacture's protocol [47]. All samples were divided into 7 groups and analyzed using separate Sentrix Array Matrices (SAMs), which accommodate 96 samples per SAM. The SNP data set was analyzed using the Illumina BeadStudio genotyping software which can cluster and call the data automatically, allowing viewing the data directly for further analysis. Those SNPs with extreme heterozygous segregation (not expected in inbred lines) or obvious errors according to the clusters were corrected manually using hybrid lines included in the study as a reference. In addition, one inbred line (CML312) was repeated in four separate plates to verify assay reproducibility. Only the most reliable calls were retained and used for further analysis, leaving us with a total of 1229 SNPs. The detailed description for data management and analysis has been reported in a previous study [33].

### Data Analysis

#### *In silico* mapping of genes

The 732 reference sequences in which the 1536 SNPs were identified were used to perform a BlastN [48] search against the maize accessioned golden path (AGP) version 1 for B73 http://www2.genome.arizona.edu/genomes/maize. Only the top blast-hits against the reference sequences were considered using an *e*-value threshold of *e* ^−18^. Blast matches to multiple loci, with the same top *e*-value were all selected for further interrogation. Of the 732 reference sequences, 13 had multiple hits to the genome and 3 matched contigs with unknown locations. A total of 1483 unique SNPs have therefore been mapped *in silico* onto the maize genome. A set of unique loci was constructed from the 1229 successfully called SNPs. The relative distance for each SNP was summed and SNPs within a total of 10 kb distance were assigned to the same locus. This was done because some genes were very long and had been sequenced in two or three amplicons; however, none of the genes were longer than 10 Kb and this distance would keep SNPs from separate amplicons from the same gene (and therefore known to be physically linked) together in the same locus. A total of 538 loci were identified. A total of 211 SNPs were unlinked to other SNPs, and the rest formed linked groups of 2 or more SNPs from contiguous DNA sequences (called loci), of which there were 327 (Table 1).

#### Population structure

The software package STUCTURE 2.2 [49] was used to investigate the population structure of the 632 lines using all 1229 successfully called SNPs, as well as the SNP loci plus SNP haplotypes. The SNPs from same locus were grouped into haplotypes that were recorded as alleles (Table 3); in this way, each locus could have multiple alleles, raising the information content of the markers. If the genotype of any SNP at a locus was missing in an individual, the locus was regarded as missing in that individual. We ran STRUCTURE testing the number of clusters (K) from 1 to 10, and each K was run 3 times with a burn-in period of 500,000 and 500,000 replications. Lines with probability of membership greater than 80% were assigned to a subgroup, while those with lower probabilities were assigned to the “mixed” subgroup.

#### Relative kinship

The relative kinship matrix comparing all pairs of the 632 lines was calculated with 700 informative SNPs with MAF>0.1 and low levels of missing data using the software package SPAGeDi [50]. Negative values between two individuals, indicating that there was less relationship than that expected between two random individuals, were changed to 0 [7].

#### Linkage disequilibrium

The linkage disequilibrium measurement parameter *r^2^* was used to estimate LD between all SNPs with less than 20% missing data on each chromosome via the software package TASSEL2.0 [46]. Linkage disequilibrium was calculated using different data sets in order to compare how different factors affect LD values. First, LD was calculated separately for all SNPs with a minor allelic frequency (MAF) less than 0.05, 0.1 and 0.2. Second, mean *r^2^* values were calculated between SNPs of different genetic distances, as described in Table 7. Third, to examine the effect of sample size on *r^2^*, 10 independent random samples of 25, 50, 100, 200 and 400 lines were used to calculate LD using SNPs with MAF greater than 0.05. Fourth, to examine the effect of subsets of tropical/subtropical and temperate groups on *r^2^*, 10 independent random samples of 80 lines were chosen from each subset to calculate LD using SNPs with MAF greater than 0.05.

#### Core set

A core set of lines was chosen to see how many lines were necessary to capture 90% of the allelic diversity present in the entire set. The analysis for the SNP haplotypes was carried out using the software package PowerMarker [51]. The simulated annealing algorithm was used to calculate the maximum number of alleles captured for a given sample size and data set. The analysis for each sample size was repeated 2500 times and the best values were reported in Tables 7 and 8.

#### Diversity comparison of tropical and temperate groups

Two major subgroups, temperate and tropical groups, were obtained based on the marker information (Table S1). To compare the diversity of the two groups, 10 random samples of 10, 20, 40, 60, 80, 100, 120 and 140 each were chosen from the two subgroups. Diversity statistics for the SNPs and SNP haplotypes for each selected sample size were calculated and compared with the subgroup and the entire dataset, respectively.

## Supporting Information

Table S1 List of the germplasm included in the study, including the pedigree name or extended pedigree for as yet-unreleased breeding lines, the probability of belonging to one of two clusters as determined by the program Structure (p A, temperate cluster, and p B, tropical cluster), and which group the line was assigned to (A, B, or mixed).
(0.75 MB DOC)
