# Supplementary material for: Genetic Characterization and Linkage Disequilibrium Estimation of a Global Maize Collection Using SNP Markers
Source: PLoS One. 2009 Dec 24;4(12):e8451. doi: 10.1371/journal.pone.0008451 (PMC2795174; doi:10.1371/journal.pone.0008451)
Supplement: Table S1 — List of the germplasm included in the study, including the pedigree name or extended pedigree for as yet-unreleased breeding lines, the probability of belonging to one of two clusters as determined by the program Structure (p A, temperate cluster, and p B, tropical cluster), and which group the line was assigned to (A, B, or mixed). (0.75 MB DOC) [file pone.0008451.s001.doc]

Table S1. List of the germplasm included in the study, including the pedigree name or extended pedigree for as yet-unreleased breeding lines, the probability of belonging to one of two clusters as determined by the program Structure (p A, temperate cluster, and p B, tropical cluster), and which group the line was assigned to (A, B, or mixed).

| **No.** | **Name** | **Pedigree** | **p A** | **p B** | **Group** |
| --- | --- | --- | --- | --- | --- |
| 1 | L2 | CML080 | 0.883 | 0.117 | B |
| 2 | L5 | CML084 | 0.837 | 0.163 | B |
| 3 | L6 | CML085 | 0.963 | 0.037 | B |
| 4 | L7 | CML088 | 0.919 | 0.081 | B |
| 5 | L8 | CML099 | 0.621 | 0.379 | Mixed |
| 6 | L9 | CML112 | 0.901 | 0.099 | B |
| 7 | L11 | CML115 | 0.767 | 0.233 | Mixed |
| 8 | L12 | CML116 | 0.779 | 0.221 | Mixed |
| 9 | L14 | CML127 | 0.939 | 0.061 | B |
| 10 | L15 | CML130 | 0.673 | 0.327 | Mixed |
| 11 | L16 | CML134 | 0.708 | 0.292 | Mixed |
| 12 | L17 | CML135 | 0.956 | 0.044 | B |
| 13 | L18 | CML139 | 0.993 | 0.007 | B |
| 14 | L19 | CML173 | 0.714 | 0.286 | Mixed |
| 15 | L20 | CML174 | 0.805 | 0.195 | B |
| 16 | L21 | CML178 | 0.745 | 0.255 | Mixed |
| 17 | L22 | CML180 | 0.642 | 0.358 | Mixed |
| 18 | L23 | CML185 | 0.773 | 0.227 | Mixed |
| 19 | L24 | CML187 | 0.661 | 0.339 | Mixed |
| 20 | L25 | CML189 | 0.771 | 0.229 | Mixed |
| 21 | L27 | CML218 | 0.991 | 0.009 | B |
| 22 | L28 | CML219 | 0.98 | 0.02 | B |
| 23 | L29 | CML220 | 0.993 | 0.007 | B |
| 24 | L30 | CML221 | 0.98 | 0.02 | B |
| 25 | L31 | CML222 | 0.971 | 0.029 | B |
| 26 | L34 | CML323 | 0.698 | 0.302 | Mixed |
| 27 | L35 | CML324 | 0.739 | 0.261 | Mixed |
| 28 | L36 | CML325 | 0.637 | 0.363 | Mixed |
| 29 | L37 | CML326 | 0.691 | 0.309 | Mixed |
| 30 | L38 | CML328 | 0.875 | 0.125 | B |
| 31 | L39 | CML330 | 0.988 | 0.012 | B |
| 32 | L40 | CML333 | 0.993 | 0.007 | B |
| 33 | L41 | CML338 | 0.989 | 0.011 | B |
| 34 | L44 | CML366 | 0.984 | 0.016 | B |
| 35 | L45 | CML367 | 0.908 | 0.092 | B |
| 36 | L46 | CML368 | 0.96 | 0.04 | B |
| 37 | L47 | CML369 | 0.664 | 0.336 | Mixed |
| 38 | L48 | CML418 | 0.986 | 0.014 | B |
| 39 | L49 | CML420 | 0.984 | 0.016 | B |
| 40 | L51 | CML422 | 0.984 | 0.016 | B |
| 41 | L52 | CML423 | 0.953 | 0.047 | B |
| 42 | L54 | CML426 | 0.912 | 0.088 | B |
| 43 | L55 | CML428 | 0.815 | 0.185 | B |
| 44 | L57 | CML440 | 0.844 | 0.156 | B |
| 45 | L58 | CML470 | 0.906 | 0.094 | B |
| 46 | L59 | CML471 | 0.978 | 0.022 | B |
| 47 | L60 | CML473 | 0.978 | 0.022 | B |
| 48 | L61 | CML474 | 0.986 | 0.014 | B |
| 49 | L62 | CML485 | 0.958 | 0.042 | B |
| 50 | L64 | 6996BULK[COMPE2/P43-SR/COMPE]FS#20-1-1-B-1-B | 0.496 | 0.504 | Mixed |
| 51 | L65 | DRB-F2-60-1-1-1 | 0.74 | 0.26 | Mixed |
| 52 | L66 | INTA-F2-192-2-1-1-1 | 0.787 | 0.213 | Mixed |
| 53 | L67 | DTPWC9-F115-1-4-1-1-B | 0.848 | 0.152 | B |
| 54 | L68 | DTPWC9-F73-2-1-1-1-B | 0.785 | 0.215 | Mixed |
| 55 | L70 | DTPWC9-F16-1-4-1-1-B | 0.917 | 0.083 | B |
| 56 | L71 | DTPYC9-F69-3-1-1-2-B | 0.949 | 0.051 | B |
| 57 | L73 | DTPYC9-F143-5-4-1-2-B | 0.82 | 0.18 | B |
| 58 | L74 | DTPYC9-F74-1-1-1-1-B | 0.851 | 0.149 | B |
| 59 | L75 | DTPYC9-F134-3-1-B | 0.803 | 0.197 | B |
| 60 | L76 | DTPYC9-F125-2-8-1-1-B | 0.985 | 0.015 | B |
| 61 | L77 | DTPYC9-F13-2-1-1-1-B | 0.776 | 0.224 | Mixed |
| 62 | L78 | CL-04935(PR8549xP23C2)-5-1-3-B*14 | 0.995 | 0.005 | B |
| 63 | L79 | CL-G1624G16C23H173-1-2-B-3-2-B*7 | 0.911 | 0.089 | B |
| 64 | L80 | CL-G1839G18SeqC3-17-1-1-2-2-B*5 | 0.879 | 0.121 | B |
| 65 | L83 | Tx114 | 0.027 | 0.973 | A |
| 66 | L84 | (P69Qc3HC107-1-1#-4-2#-4-B-B-1-4-B-B-B-B-BXCML193)-B-B-2-B-B-B | 0.721 | 0.279 | Mixed |
| 67 | L85 | Pop.69TempladoAmarilloQPM-B-B-B1-6-B-B-B | 0.683 | 0.317 | Mixed |
| 68 | L86 | Pop.70TempladoAmarilloDentadoQPM-B-B-B2-8-B-B-B | 0.741 | 0.259 | Mixed |
| 69 | L91 | (NC258/NC300)-B-2-2-B-B | 0.567 | 0.433 | Mixed |
| 70 | L95 | ((B104/NC300)x(CML415/B104))-4-2-B-B | 0.394 | 0.606 | Mixed |
| 71 | L96 | ((B104/NC300)x(CML285/B104))-2-3-B-B | 0.461 | 0.539 | Mixed |
| 72 | L99 | (CML326/B104)-B-9-B-B-B-B | 0.311 | 0.689 | Mixed |
| 73 | L101 | (CML288/NC300)-B-9-B1-B-B-B | 0.991 | 0.009 | B |
| 74 | L102 | (NC300/Tx772)-B-1-B2-B-B | 0.989 | 0.011 | B |
| 75 | L108 | LAMA2002-46-2-B | 0.9 | 0.1 | B |
| 76 | L109 | LAMA2002-53-5-B | 0.978 | 0.022 | B |
| 77 | L110 | LAMA2002-58-4-B | 0.989 | 0.011 | B |
| 78 | L111 | LAMA2002-60-9-B | 0.951 | 0.049 | B |
| 79 | L112 | (Tx601xB104-B/B110xFR2128-B)-B-B-4-B-B-B | 0.187 | 0.813 | A |
| 80 | L113 | Pop.147-F2#132-1-1-B-2-B-B-B-B | 0.987 | 0.013 | B |
| 81 | L114 | P84c3BcxLLTtardAsiaxMIRTF41-2-1-2-2-B-B | 0.882 | 0.118 | B |
| 82 | L115 | CTS013174/SW1(S)C11-42-1-B-B-1-3-BBBB/Nei9202-B | 0.993 | 0.007 | B |
| 83 | L116 | Nei9008-B-B-B | 0.988 | 0.012 | B |
| 84 | L117 | Nei402011-B-B-B | 0.954 | 0.046 | B |
| 85 | L119 | Nei402025-B-B-B | 0.915 | 0.085 | B |
| 86 | L120 | Nei402026-B-B-B | 0.865 | 0.135 | B |
| 87 | L121 | CML004 | 0.976 | 0.024 | B |
| 88 | L122 | CML020 | 0.989 | 0.011 | B |
| 89 | L123 | CML022 | 0.96 | 0.04 | B |
| 90 | L124 | CML023 | 0.994 | 0.006 | B |
| 91 | L125 | CML026 | 0.955 | 0.045 | B |
| 92 | L126 | CML029 | 0.922 | 0.078 | B |
| 93 | L127 | CML031 | 0.976 | 0.024 | B |
| 94 | L128 | CML035 | 0.99 | 0.01 | B |
| 95 | L129 | CML037 | 0.995 | 0.005 | B |
| 96 | L130 | CML039 | 0.993 | 0.007 | B |
| 97 | L131 | CML040 | 0.908 | 0.092 | B |
| 98 | L133 | CML050 | 0.983 | 0.017 | B |
| 99 | L136 | CML068 | 0.995 | 0.005 | B |
| 100 | L137 | CML069 | 0.992 | 0.008 | B |
| 101 | L138 | CML086 | 0.991 | 0.009 | B |
| 102 | L139 | CML087 | 0.892 | 0.108 | B |
| 103 | L140 | CML089 | 0.985 | 0.015 | B |
| 104 | L141 | CML091 | 0.988 | 0.012 | B |
| 105 | L142 | CML092 | 0.782 | 0.218 | Mixed |
| 106 | L143 | CML094 | 0.918 | 0.082 | B |
| 107 | L144 | CML095 | 0.965 | 0.035 | B |
| 108 | L145 | CML096 | 0.808 | 0.192 | B |
| 109 | L146 | CML097 | 0.75 | 0.25 | Mixed |
| 110 | L147 | CML098 | 0.738 | 0.262 | Mixed |
| 111 | L149 | CML101 | 0.843 | 0.157 | B |
| 112 | L150 | CML103 | 0.965 | 0.035 | B |
| 113 | L151 | CML107 | 0.991 | 0.009 | B |
| 114 | L152 | CML108 | 0.952 | 0.048 | B |
| 115 | L153 | CML111 | 0.897 | 0.103 | B |
| 116 | L154 | CML114 | 0.757 | 0.243 | Mixed |
| 117 | L155 | CML118 | 0.964 | 0.036 | B |
| 118 | L156 | CML121 | 0.989 | 0.011 | B |
| 119 | L157 | CML122 | 0.988 | 0.012 | B |
| 120 | L158 | CML123 | 0.989 | 0.011 | B |
| 121 | L159 | CML129 | 0.974 | 0.026 | B |
| 122 | L160 | CML132 | 0.993 | 0.007 | B |
| 123 | L161 | CML138 | 0.988 | 0.012 | B |
| 124 | L162 | CML140 | 0.992 | 0.008 | B |
| 125 | L163 | CML141 | 0.991 | 0.009 | B |
| 126 | L164 | CML154 | 0.963 | 0.037 | B |
| 127 | L165 | CML157 | 0.991 | 0.009 | B |
| 128 | L167 | CML161 | 0.906 | 0.094 | B |
| 129 | L168 | CML162 | 0.928 | 0.072 | B |
| 130 | L169 | CML163 | 0.923 | 0.077 | B |
| 131 | L170 | CML166 | 0.934 | 0.066 | B |
| 132 | L171 | CML168 | 0.98 | 0.02 | B |
| 133 | L172 | CML169 | 0.964 | 0.036 | B |
| 134 | L173 | CML170 | 0.899 | 0.101 | B |
| 135 | L174 | CML171 | 0.994 | 0.006 | B |
| 136 | L175 | CML172 | 0.993 | 0.007 | B |
| 137 | L176 | CML175 | 0.937 | 0.063 | B |
| 138 | L178 | CML182 | 0.413 | 0.587 | Mixed |
| 139 | L179 | CML186 | 0.669 | 0.331 | Mixed |
| 140 | L180 | CML191 | 0.639 | 0.361 | Mixed |
| 141 | L181 | CML192 | 0.667 | 0.333 | Mixed |
| 142 | L185 | CML223 | 0.992 | 0.008 | B |
| 143 | L187 | CML225 | 0.985 | 0.015 | B |
| 144 | L188 | CML226 | 0.945 | 0.055 | B |
| 145 | L189 | CML228 | 0.987 | 0.013 | B |
| 146 | L190 | CML229 | 0.965 | 0.035 | B |
| 147 | L191 | CML230 | 0.983 | 0.017 | B |
| 148 | L192 | CML231 | 0.982 | 0.018 | B |
| 149 | L193 | CML232 | 0.954 | 0.046 | B |
| 150 | L196 | CML249 | 0.977 | 0.023 | B |
| 151 | L197 | CML259 | 0.993 | 0.007 | B |
| 152 | L198 | CML261 | 0.994 | 0.006 | B |
| 153 | L199 | CML269 | 0.988 | 0.012 | B |
| 154 | L200 | CML282 | 0.921 | 0.079 | B |
| 155 | L201 | CML283 | 0.973 | 0.027 | B |
| 156 | L202 | CML286 | 0.985 | 0.015 | B |
| 157 | L204 | CML306 | 0.991 | 0.009 | B |
| 158 | L205 | CML310 | 0.99 | 0.01 | B |
| 159 | L208 | CML316 | 0.795 | 0.205 | Mixed |
| 160 | L209 | CML317 | 0.888 | 0.112 | B |
| 161 | L210 | CML321 | 0.975 | 0.025 | B |
| 162 | L211 | CML322 | 0.991 | 0.009 | B |
| 163 | L213 | CML331 | 0.994 | 0.006 | B |
| 164 | L214 | CML334 | 0.967 | 0.033 | B |
| 165 | L216 | CML360 | 0.992 | 0.008 | B |
| 166 | L217 | CML361 | 0.991 | 0.009 | B |
| 167 | L220 | CML371 | 0.977 | 0.023 | B |
| 168 | L221 | CML372 | 0.841 | 0.159 | B |
| 169 | L222 | CML373 | 0.913 | 0.087 | B |
| 170 | L223 | CML374 | 0.992 | 0.008 | B |
| 171 | L224 | CML376 | 0.955 | 0.045 | B |
| 172 | L225 | CML379 | 0.992 | 0.008 | B |
| 173 | L226 | CML381 | 0.989 | 0.011 | B |
| 174 | L227 | CML382 | 0.994 | 0.006 | B |
| 175 | L229 | CML411 | 0.973 | 0.027 | B |
| 176 | L230 | CML416 | 0.992 | 0.008 | B |
| 177 | L231 | CML431 | 0.898 | 0.102 | B |
| 178 | L232 | CML432 | 0.954 | 0.046 | B |
| 179 | L233 | CML450 | 0.952 | 0.048 | B |
| 180 | L234 | CML451 | 0.964 | 0.036 | B |
| 181 | L235 | CML465 | 0.991 | 0.009 | B |
| 182 | L240 | CML479 | 0.995 | 0.005 | B |
| 183 | L242 | CML482 | 0.956 | 0.044 | B |
| 184 | L243 | CML483 | 0.975 | 0.025 | B |
| 185 | L244 | CML484 | 0.983 | 0.017 | B |
| 186 | L245 | CML490 | 0.993 | 0.007 | B |
| 187 | L246 | CML493 | 0.978 | 0.022 | B |
| 188 | L247 | CML495 | 0.99 | 0.01 | B |
| 189 | L248 | P1 | 0.992 | 0.008 | B |
| 190 | L249 | H-16 | 0.991 | 0.009 | B |
| 191 | L250 | DTP2WC4H255-1-2-2-BB/LATA-F2-138 | 0.9 | 0.1 | B |
| 192 | L252 | DTPWC8F347-1-3-1-B | 0.906 | 0.094 | B |
| 193 | L253 | DTPWC8F324-1-1-1-#-B | 0.809 | 0.191 | B |
| 194 | L254 | DTPWC8F266-1-1-1-#-B | 0.985 | 0.015 | B |
| 195 | L255 | DTPWC9-F109-2-6-1-1-B | 0.829 | 0.171 | B |
| 196 | L256 | DTPWC9-F2-3-2-1-1-B | 0.98 | 0.02 | B |
| 197 | L257 | DTPWC9-F103-2-1-1-1-B | 0.984 | 0.016 | B |
| 198 | L258 | DTPWC9-F18-1-3-1-1-B | 0.912 | 0.088 | B |
| 199 | L259 | DTPYC9-F11-2-3-1-2-B | 0.765 | 0.235 | Mixed |
| 200 | L260 | DTPYC9-F46-3-9-1-2-B | 0.955 | 0.045 | B |
| 201 | L261 | DTPYC9-F65-2-3-1-1-B | 0.965 | 0.035 | B |
| 202 | L262 | DTPYC9-F116-2-1-1-1-B | 0.895 | 0.105 | B |
| 203 | L263 | DTPYC9-F38-4-6-1-1-B | 0.838 | 0.162 | B |
| 204 | L264 | LaPostaSeq.C0F12-2-1-1 | 0.989 | 0.011 | B |
| 205 | L265 | CL-02841Ac8928-40-1-1-1-1-B | 0.981 | 0.019 | B |
| 206 | L266 | CL-04930(P49C2H12-1-4xPR8549-1-1)-1-1-3-B*8 | 0.995 | 0.005 | B |
| 207 | L267 | CL-04934(P49C2H12-5-4xP23C2-11-1)-2-2-2-B*10 | 0.996 | 0.004 | B |
| 208 | L268 | CL-G1632G16C20MH44-#-3-3-1-B*5 | 0.912 | 0.088 | B |
| 209 | L271 | CL-G2606G26SeqC1-149-1-1-2-1-2-1-BB | 0.98 | 0.02 | B |
| 210 | L272 | CL-G2609G26C23-75-1-1-2-1-B*5 | 0.976 | 0.024 | B |
| 211 | L273 | CL-G2611G26SEQF135-3-3-1-1-1-2-BB | 0.981 | 0.019 | B |
| 212 | L275 | CL-QRCWQ15 | 0.986 | 0.014 | B |
| 213 | L277 | CL-SCBY03 | 0.976 | 0.024 | B |
| 214 | L278 | CL-SPLW05SPLC7F275-1-1-1-1-1-B-B | 0.985 | 0.015 | B |
| 215 | L280 | ((Ko326yxTx806)-6-1-1-1-B-B/CML161)x(Tx802/CML161))-1-B-B-B | 0.513 | 0.487 | Mixed |
| 216 | L284 | ((CML408/B104)x(CML411/B104))-1-1-B-B | 0.459 | 0.541 | Mixed |
| 217 | L285 | ((CML285/B104)x(CML288/NC300))-2-1-B-B | 0.437 | 0.563 | Mixed |
| 218 | L286 | (CML285/NC300)-B-6-B-B-B-B | 0.976 | 0.024 | B |
| 219 | L289 | LAMA2002-10-1-B | 0.985 | 0.015 | B |
| 220 | L290 | LAMA2002-20-6-B | 0.995 | 0.005 | B |
| 221 | L291 | LAMA2002-43-2-B | 0.99 | 0.01 | B |
| 222 | L292 | LAMA2002-61-1-B | 0.973 | 0.027 | B |
| 223 | L293 | MBRC6AmF9-2-B-#-3-1-B-B-B-B-B | 0.982 | 0.018 | B |
| 224 | L294 | CML329/MBRC3AmF25-2-1-1-B-B-B | 0.836 | 0.164 | B |
| 225 | L296 | SRR-C1SA3MH32-4-3-B-1-1-2-B-B | 0.966 | 0.034 | B |
| 226 | L297 | P390Am/CMLc4F253-B-2-2-4-1-B-B | 0.972 | 0.028 | B |
| 227 | L298 | AMATLC0HS71-1-1-2-1-1-1-BBBB-B-B-B | 0.991 | 0.009 | B |
| 228 | L299 | Pop.28C9HC113-3-1-4-B*8-B-B-B | 0.994 | 0.006 | B |
| 229 | L300 | P84c3BcxP391c3F38-1-3-2-2-1-1-2-B-B | 0.993 | 0.007 | B |
| 230 | L301 | P84c3BcxLinerecycleLLTpreAsiaxMIRTF59-2-1-1-1-B-B | 0.988 | 0.012 | B |
| 231 | L302 | MIRTC4AmF17-B-2-1-B-B | 0.991 | 0.009 | B |
| 232 | L303 | P391c2F22-1-1-2-1-B-B-B | 0.988 | 0.012 | B |
| 233 | L304 | MBRC6BcF234-1-B-#-1-1-B-B-B-B-B | 0.982 | 0.018 | B |
| 234 | L305 | MBR-ET(W)C1F139-2-1-B-2-B-B-B-B-B-BxMBRC5BcF13-3-1-2-B-B-B-B-1-2-B-B-B | 0.971 | 0.029 | B |
| 235 | L309 | Ejura/Sin35C4F52-2-2-2-B-B-B | 0.92 | 0.08 | B |
| 236 | L310 | Cuba/GuadC3F53-3-1-1-B-B-B | 0.978 | 0.022 | B |
| 237 | L311 | P591c4F3-1-2-2-B-B-B | 0.974 | 0.026 | B |
| 238 | L312 | P591c4F55-2-2-2-B-B-B | 0.966 | 0.034 | B |
| 239 | L313 | MIRTC5BcoF62-2-2-1-1-2-1-B-B | 0.974 | 0.026 | B |
| 240 | L314 | Pob.391C4F91-1-2-1-B-B | 0.959 | 0.041 | B |
| 241 | L316 | P84c3BcxMIRTC5BcoF10-1-2-2-2-3-1-B-B | 0.983 | 0.017 | B |
| 242 | L317 | P84c3BcxMIRTC5BcoF80-4-2-1-4-1-1-B-B | 0.993 | 0.007 | B |
| 243 | L318 | P84c3BcxP390Bco/CMLc4F92-B-2-1-2-1-B-B | 0.987 | 0.013 | B |
| 244 | L321 | CML003 | 0.992 | 0.008 | B |
| 245 | L322 | CML005 | 0.996 | 0.004 | B |
| 246 | L324 | CML009 | 0.989 | 0.011 | B |
| 247 | L325 | CML012 | 0.99 | 0.01 | B |
| 248 | L326 | CML013 | 0.993 | 0.007 | B |
| 249 | L328 | CML015 | 0.995 | 0.005 | B |
| 250 | L329 | CML016 | 0.989 | 0.011 | B |
| 251 | L330 | CML017 | 0.984 | 0.016 | B |
| 252 | L331 | CML024 | 0.992 | 0.008 | B |
| 253 | L332 | CML027 | 0.978 | 0.022 | B |
| 254 | L333 | CML028 | 0.995 | 0.005 | B |
| 255 | L334 | CML032 | 0.993 | 0.007 | B |
| 256 | L335 | CML038 | 0.991 | 0.009 | B |
| 257 | L337 | CML043 | 0.953 | 0.047 | B |
| 258 | L340 | CML047 | 0.989 | 0.011 | B |
| 259 | L341 | CML048 | 0.993 | 0.007 | B |
| 260 | L342 | CML049 | 0.991 | 0.009 | B |
| 261 | L343 | CML051 | 0.994 | 0.006 | B |
| 262 | L349 | CML133 | 0.641 | 0.359 | Mixed |
| 263 | L350 | CML142 | 0.872 | 0.128 | B |
| 264 | L351 | CML144 | 0.936 | 0.064 | B |
| 265 | L352 | CML147 | 0.942 | 0.058 | B |
| 266 | L353 | CML150 | 0.901 | 0.099 | B |
| 267 | L354 | CML153 | 0.989 | 0.011 | B |
| 268 | L355 | CML158 | 0.973 | 0.027 | B |
| 269 | L356 | CML159 | 0.99 | 0.01 | B |
| 270 | L357 | CML165 | 0.971 | 0.029 | B |
| 271 | L358 | CML196 | 0.799 | 0.201 | Mixed |
| 272 | L360 | CML201 | 0.958 | 0.042 | B |
| 273 | L361 | CML202 | 0.801 | 0.199 | B |
| 274 | L362 | CML204 | 0.863 | 0.137 | B |
| 275 | L363 | CML206 | 0.952 | 0.048 | B |
| 276 | L364 | CML208 | 0.865 | 0.135 | B |
| 277 | L365 | CML216 | 0.743 | 0.257 | Mixed |
| 278 | L366 | CML238 | 0.993 | 0.007 | B |
| 279 | L368 | CML248 | 0.954 | 0.046 | B |
| 280 | L370 | CML254 | 0.992 | 0.008 | B |
| 281 | L371 | CML257 | 0.993 | 0.007 | B |
| 282 | L372 | CML260 | 0.993 | 0.007 | B |
| 283 | L373 | CML268 | 0.995 | 0.005 | B |
| 284 | L374 | CML270 | 0.988 | 0.012 | B |
| 285 | L377 | CML274 | 0.989 | 0.011 | B |
| 286 | L379 | CML279 | 0.994 | 0.006 | B |
| 287 | L380 | CML281 | 0.972 | 0.028 | B |
| 288 | L381 | CML285 | 0.991 | 0.009 | B |
| 289 | L382 | CML287 | 0.967 | 0.033 | B |
| 290 | L383 | CML289 | 0.99 | 0.01 | B |
| 291 | L384 | CML290 | 0.991 | 0.009 | B |
| 292 | L386 | CML298 | 0.992 | 0.008 | B |
| 293 | L387 | CML307 | 0.994 | 0.006 | B |
| 294 | L388 | CML319 | 0.964 | 0.036 | B |
| 295 | L389 | CML320 | 0.829 | 0.171 | B |
| 296 | L390 | CML327 | 0.57 | 0.43 | Mixed |
| 297 | L392 | CML340 | 0.993 | 0.007 | B |
| 298 | L393 | CML341 | 0.993 | 0.007 | B |
| 299 | L394 | CML343 | 0.994 | 0.006 | B |
| 300 | L395 | CML344 | 0.987 | 0.013 | B |
| 301 | L397 | CML364 | 0.993 | 0.007 | B |
| 302 | L398 | CML377 | 0.97 | 0.03 | B |
| 303 | L399 | CML378 | 0.996 | 0.004 | B |
| 304 | L400 | CML384 | 0.991 | 0.009 | B |
| 305 | L401 | CML386 | 0.81 | 0.19 | B |
| 306 | L402 | CML387 | 0.955 | 0.045 | B |
| 307 | L403 | CML389 | 0.988 | 0.012 | B |
| 308 | L405 | CML395 | 0.834 | 0.166 | B |
| 309 | L406 | CML397 | 0.982 | 0.018 | B |
| 310 | L408 | CML401 | 0.943 | 0.057 | B |
| 311 | L409 | CML402 | 0.977 | 0.023 | B |
| 312 | L412 | CML405 | 0.982 | 0.018 | B |
| 313 | L413 | CML406 | 0.994 | 0.006 | B |
| 314 | L414 | CML407 | 0.988 | 0.012 | B |
| 315 | L415 | CML408 | 0.986 | 0.014 | B |
| 316 | L416 | CML412 | 0.99 | 0.01 | B |
| 317 | L417 | CML413 | 0.99 | 0.01 | B |
| 318 | L418 | CML415 | 0.949 | 0.051 | B |
| 319 | L419 | CML430 | 0.987 | 0.013 | B |
| 320 | L420 | CML433 | 0.888 | 0.112 | B |
| 321 | L421 | CML442 | 0.785 | 0.215 | Mixed |
| 322 | L423 | CML446 | 0.981 | 0.019 | B |
| 323 | L426 | CML454 | 0.994 | 0.006 | B |
| 324 | L428 | CML468 | 0.994 | 0.006 | B |
| 325 | L429 | CML476 | 0.971 | 0.029 | B |
| 326 | L430 | CML494 | 0.993 | 0.007 | B |
| 327 | L431 | CML496 | 0.993 | 0.007 | B |
| 328 | L432 | CML497 | 0.994 | 0.006 | B |
| 329 | L433 | P2 | 0.973 | 0.027 | B |
| 330 | L436 | CML-395/CML444B-4-1-3-1 | 0.953 | 0.047 | B |
| 331 | L437 | SW1SR/COMPE1-W###52#-19-5-1-B*5 | 0.49 | 0.51 | Mixed |
| 332 | L438 | DTPWC8F317-1-1-1-#-B | 0.77 | 0.23 | Mixed |
| 333 | L439 | LaPostaSeq.C4F140-1-1-1 | 0.986 | 0.014 | B |
| 334 | L440 | LaPostaSeq.C4F273-2-2-1 | 0.995 | 0.005 | B |
| 335 | L441 | CL-04343 | 0.994 | 0.006 | B |
| 336 | L442 | CL-Q6203 | 0.972 | 0.028 | B |
| 337 | L443 | CL-RCW01 | 0.989 | 0.011 | B |
| 338 | L445 | MBR/MDRAmC4F55-2-B-#-1-1-B-B-B-B-B | 0.991 | 0.009 | B |
| 339 | L446 | 1760AA1Amx1751Ay52AComp.-B-1-2-B-B-B-B-B-B | 0.986 | 0.014 | B |
| 340 | L447 | 761BB2Bcox751B-B-1-1-B-B-B-B-B-B | 0.994 | 0.006 | B |
| 341 | L448 | Cuba/GuadC3F42-2-1-1-B-B-B | 0.988 | 0.012 | B |
| 342 | L449 | Cuba/GuadC3F125-2-2-1-B-B-B | 0.988 | 0.012 | B |
| 343 | L450 | P591c41y2GENF3-1-1-2-B-B-B | 0.983 | 0.017 | B |
| 344 | L451 | P591c41y2GENF205-1-1-1-B-B-B | 0.99 | 0.01 | B |
| 345 | L452 | MIRTC5AmF24-2-1-1-3-1-2-B-B | 0.952 | 0.048 | B |
| 346 | L453 | MBR/MDRC4BcF34-1-B-#-1-1-B-B-B-B-B | 0.993 | 0.007 | B |
| 347 | L454 | MBR/MDRC3Bc/MBRC5BcF59-1-B-#-1-2-B-B-B-B-B | 0.892 | 0.108 | B |
| 348 | L458 | Cuba/GuadC3F110-2-2-1-B-B-B | 0.983 | 0.017 | B |
| 349 | L459 | P590C7BlancosF57-1-3-1-B-B-B | 0.937 | 0.063 | B |
| 350 | L460 | P391c4BcoF105-1-2-3-B-B | 0.994 | 0.006 | B |
| 351 | CML312 | CML312 | 0.993 | 0.007 | B |
| 352 | U1 | 4226 | 0.005 | 0.995 | A |
| 353 | U2 | 4722 | 0.034 | 0.966 | A |
| 354 | U3 | 38-11 | 0.019 | 0.981 | A |
| 355 | U4 | 33-16 | 0.05 | 0.95 | A |
| 356 | U5 | A188 | 0.354 | 0.646 | Mixed |
| 357 | U6 | A214N | 0.167 | 0.833 | A |
| 358 | U7 | A239 | 0.015 | 0.985 | A |
| 359 | U8 | A272 | 0.6 | 0.4 | Mixed |
| 360 | U9 | A441-5 | 0.209 | 0.791 | Mixed |
| 361 | U10 | A554 | 0.016 | 0.984 | A |
| 362 | U11 | A556 | 0.12 | 0.88 | A |
| 363 | U12 | A6 | 0.995 | 0.005 | B |
| 364 | U13 | A619 | 0.01 | 0.99 | A |
| 365 | U14 | A632 | 0.005 | 0.995 | A |
| 366 | U15 | A634 | 0.006 | 0.994 | A |
| 367 | U16 | A635 | 0.006 | 0.994 | A |
| 368 | U17 | A641 | 0.004 | 0.996 | A |
| 369 | U18 | A654 | 0.014 | 0.986 | A |
| 370 | U19 | A659 | 0.005 | 0.995 | A |
| 371 | U20 | A661 | 0.01 | 0.99 | A |
| 372 | U21 | A679 | 0.004 | 0.996 | A |
| 373 | U22 | A680 | 0.003 | 0.997 | A |
| 374 | U23 | A682 | 0.008 | 0.992 | A |
| 375 | U24 | Ab28A | 0.216 | 0.784 | Mixed |
| 376 | U25 | B10 | 0.004 | 0.996 | A |
| 377 | U26 | B103 | 0.014 | 0.986 | A |
| 378 | U27 | B104 | 0.003 | 0.997 | A |
| 379 | U28 | B105 | 0.005 | 0.995 | A |
| 380 | U29 | B109 | 0.003 | 0.997 | A |
| 381 | U30 | B115 | 0.032 | 0.968 | A |
| 382 | U31 | B14A | 0.004 | 0.996 | A |
| 383 | U32 | B164 | 0.075 | 0.925 | A |
| 384 | U33 | B2 | 0.008 | 0.992 | A |
| 385 | U34 | B37 | 0.006 | 0.994 | A |
| 386 | U35 | B46 | 0.009 | 0.991 | A |
| 387 | U36 | B52 | 0.013 | 0.987 | A |
| 388 | U37 | B57 | 0.254 | 0.746 | Mixed |
| 389 | U38 | B64 | 0.056 | 0.944 | A |
| 390 | U39 | B68 | 0.012 | 0.988 | A |
| 391 | U40 | B73 | 0.003 | 0.997 | A |
| 392 | U41 | B73Htrhm | 0.003 | 0.997 | A |
| 393 | U42 | B75 | 0.017 | 0.983 | A |
| 394 | U43 | B76 | 0.016 | 0.984 | A |
| 395 | U44 | B77 | 0.038 | 0.962 | A |
| 396 | U45 | B79 | 0.035 | 0.965 | A |
| 397 | U46 | B84 | 0.005 | 0.995 | A |
| 398 | U47 | B97 | 0.04 | 0.96 | A |
| 399 | U48 | C103 | 0.019 | 0.981 | A |
| 400 | U49 | C123 | 0.019 | 0.981 | A |
| 401 | U50 | C49A | 0.036 | 0.964 | A |
| 402 | U51 | CH701-30 | 0.054 | 0.946 | A |
| 403 | U52 | CH9 | 0.172 | 0.828 | A |
| 404 | U53 | CI.7 | 0.008 | 0.992 | A |
| 405 | U54 | CI187-2 | 0.411 | 0.589 | Mixed |
| 406 | U55 | CI21E | 0.014 | 0.986 | A |
| 407 | U56 | CI28A | 0.279 | 0.721 | Mixed |
| 408 | U57 | CI31A | 0.12 | 0.88 | A |
| 409 | U58 | CI3A | 0.067 | 0.933 | A |
| 410 | U59 | CI64 | 0.464 | 0.536 | Mixed |
| 411 | U60 | CI66 | 0.288 | 0.712 | Mixed |
| 412 | U61 | CI90C | 0.13 | 0.87 | A |
| 413 | U62 | CI91B | 0.005 | 0.995 | A |
| 414 | U63 | CM105 | 0.004 | 0.996 | A |
| 415 | U64 | CM174 | 0.004 | 0.996 | A |
| 416 | U65 | CM37 | 0.008 | 0.992 | A |
| 417 | U66 | CM7 | 0.015 | 0.985 | A |
| 418 | U67 | CML10 | 0.977 | 0.023 | B |
| 419 | U68 | CML103 | 0.976 | 0.024 | B |
| 420 | U69 | CML108 | 0.947 | 0.053 | B |
| 421 | U70 | CML11 | 0.994 | 0.006 | B |
| 422 | U71 | CML14 | 0.987 | 0.013 | B |
| 423 | U72 | CML154Q | 0.982 | 0.018 | B |
| 424 | U73 | CML157Q | 0.991 | 0.009 | B |
| 425 | U74 | CML158Q | 0.982 | 0.018 | B |
| 426 | U75 | CML218 | 0.987 | 0.013 | B |
| 427 | U76 | CML220 | 0.91 | 0.09 | B |
| 428 | U77 | CML228 | 0.99 | 0.01 | B |
| 429 | U78 | CML238 | 0.994 | 0.006 | B |
| 430 | U79 | CML247 | 0.061 | 0.939 | A |
| 431 | U80 | CML254 | 0.991 | 0.009 | B |
| 432 | U81 | CML258 | 0.993 | 0.007 | B |
| 433 | U82 | CML261 | 0.993 | 0.007 | B |
| 434 | U83 | CML264 | 0.937 | 0.063 | B |
| 435 | U84 | CML277 | 0.988 | 0.012 | B |
| 436 | U85 | CML281 | 0.983 | 0.017 | B |
| 437 | U86 | CML287 | 0.957 | 0.043 | B |
| 438 | U87 | CML311 | 0.97 | 0.03 | B |
| 439 | U88 | CML314 | 0.993 | 0.007 | B |
| 440 | U89 | CML321 | 0.979 | 0.021 | B |
| 441 | U90 | CML322 | 0.878 | 0.122 | B |
| 442 | U91 | CML323 | 0.695 | 0.305 | Mixed |
| 443 | U92 | CML328 | 0.698 | 0.302 | Mixed |
| 444 | U93 | CML331 | 0.991 | 0.009 | B |
| 445 | U94 | CML332 | 0.994 | 0.006 | B |
| 446 | U95 | CML333 | 0.991 | 0.009 | B |
| 447 | U96 | CML341 | 0.991 | 0.009 | B |
| 448 | U97 | CML38 | 0.979 | 0.021 | B |
| 449 | U98 | CML45 | 0.994 | 0.006 | B |
| 450 | U99 | CML5 | 0.996 | 0.004 | B |
| 451 | U100 | CML52 | 0.991 | 0.009 | B |
| 452 | U101 | CML61 | 0.992 | 0.008 | B |
| 453 | U102 | CML69 | 0.983 | 0.017 | B |
| 454 | U103 | CML77 | 0.809 | 0.191 | B |
| 455 | U104 | CML91 | 0.973 | 0.027 | B |
| 456 | U105 | CML92 | 0.752 | 0.248 | Mixed |
| 457 | U106 | CMV3 | 0.022 | 0.978 | A |
| 458 | U107 | CO106 | 0.106 | 0.894 | A |
| 459 | U108 | CO125 | 0.086 | 0.914 | A |
| 460 | U109 | CO255 | 0.146 | 0.854 | A |
| 461 | U110 | D940Y | 0.286 | 0.714 | Mixed |
| 462 | U111 | DE_2 | 0.049 | 0.951 | A |
| 463 | U112 | DE_3 | 0.01 | 0.99 | A |
| 464 | U113 | DE1 | 0.039 | 0.961 | A |
| 465 | U114 | DE811 | 0.054 | 0.946 | A |
| 466 | U115 | E2558W | 0.419 | 0.581 | Mixed |
| 467 | U116 | EP1 | 0.276 | 0.724 | Mixed |
| 468 | U117 | F2834T | 0.658 | 0.342 | Mixed |
| 469 | U118 | F44 | 0.374 | 0.626 | Mixed |
| 470 | U119 | F6 | 0.208 | 0.792 | Mixed |
| 471 | U120 | F7 | 0.201 | 0.799 | Mixed |
| 472 | U121 | GA209 | 0.231 | 0.769 | Mixed |
| 473 | U122 | GT112 | 0.437 | 0.563 | Mixed |
| 474 | U123 | H105W | 0.007 | 0.993 | A |
| 475 | U124 | H49 | 0.158 | 0.842 | A |
| 476 | U125 | H84 | 0.005 | 0.995 | A |
| 477 | U126 | H91 | 0.004 | 0.996 | A |
| 478 | U127 | H95 | 0.039 | 0.961 | A |
| 479 | U128 | H99 | 0.043 | 0.957 | A |
| 480 | U129 | Hi27 | 0.822 | 0.178 | B |
| 481 | U130 | HP301 | 0.036 | 0.964 | A |
| 482 | U131 | Hy | 0.008 | 0.992 | A |
| 483 | U132 | I137TN | 0.506 | 0.494 | Mixed |
| 484 | U133 | I205 | 0.07 | 0.93 | A |
| 485 | U134 | I29 | 0.246 | 0.754 | Mixed |
| 486 | U135 | IA2132 | 0.035 | 0.965 | A |
| 487 | U136 | Ia5125 | 0.011 | 0.989 | A |
| 488 | U137 | IDS28 | 0.15 | 0.85 | A |
| 489 | U138 | IDS69 | 0.032 | 0.968 | A |
| 490 | U139 | IDS91 | 0.022 | 0.978 | A |
| 491 | U140 | Il101 | 0.006 | 0.994 | A |
| 492 | U141 | Il14H | 0.01 | 0.99 | A |
| 493 | U142 | Il677a | 0.078 | 0.922 | A |
| 494 | U143 | K148 | 0.203 | 0.797 | Mixed |
| 495 | U144 | K4 | 0.056 | 0.944 | A |
| 496 | U145 | K55 | 0.078 | 0.922 | A |
| 497 | U146 | K64 | 0.25 | 0.75 | Mixed |
| 498 | U147 | Ki11 | 0.973 | 0.027 | B |
| 499 | U148 | Ki14 | 0.986 | 0.014 | B |
| 500 | U149 | Ki2021 | 0.983 | 0.017 | B |
| 501 | U150 | Ki21 | 0.624 | 0.376 | Mixed |
| 502 | U151 | Ki3 | 0.988 | 0.012 | B |
| 503 | U152 | Ki43 | 0.932 | 0.068 | B |
| 504 | U153 | Ki44 | 0.966 | 0.034 | B |
| 505 | U154 | Ky21 | 0.028 | 0.972 | A |
| 506 | U155 | KY226 | 0.373 | 0.627 | Mixed |
| 507 | U156 | KY228 | 0.154 | 0.846 | A |
| 508 | U157 | L317 | 0.243 | 0.757 | Mixed |
| 509 | U158 | L578 | 0.497 | 0.503 | Mixed |
| 510 | U159 | M14 | 0.007 | 0.993 | A |
| 511 | U160 | M162W | 0.367 | 0.633 | Mixed |
| 512 | U161 | M37W | 0.664 | 0.336 | Mixed |
| 513 | U162 | MEF156-5 | 0.01 | 0.99 | A |
| 514 | U163 | Mo17 | 0.005 | 0.995 | A |
| 515 | U164 | Mo18W | 0.931 | 0.069 | B |
| 516 | U165 | MO1W | 0.132 | 0.868 | A |
| 517 | U166 | Mo24W | 0.441 | 0.559 | Mixed |
| 518 | U167 | Mo44 | 0.021 | 0.979 | A |
| 519 | U168 | Mo45 | 0.156 | 0.844 | A |
| 520 | U169 | Mo46 | 0.137 | 0.863 | A |
| 521 | U170 | Mo47 | 0.217 | 0.783 | Mixed |
| 522 | U171 | MoG | 0.225 | 0.775 | Mixed |
| 523 | U172 | Mp339 | 0.249 | 0.751 | Mixed |
| 524 | U173 | MS1334 | 0.378 | 0.622 | Mixed |
| 525 | U174 | MS153 | 0.012 | 0.988 | A |
| 526 | U175 | MS71 | 0.01 | 0.99 | A |
| 527 | U176 | Mt42 | 0.077 | 0.923 | A |
| 528 | U177 | N192 | 0.006 | 0.994 | A |
| 529 | U178 | N28Ht | 0.006 | 0.994 | A |
| 530 | U179 | N6 | 0.08 | 0.92 | A |
| 531 | U180 | N7A | 0.012 | 0.988 | A |
| 532 | U181 | NC222 | 0.316 | 0.684 | Mixed |
| 533 | U182 | NC230 | 0.176 | 0.824 | A |
| 534 | U183 | NC232 | 0.184 | 0.816 | A |
| 535 | U184 | NC236 | 0.301 | 0.699 | Mixed |
| 536 | U185 | NC238 | 0.309 | 0.691 | Mixed |
| 537 | U186 | NC250 | 0.134 | 0.866 | A |
| 538 | U187 | NC258 | 0.01 | 0.99 | A |
| 539 | U188 | NC260 | 0.066 | 0.934 | A |
| 540 | U189 | NC262 | 0.015 | 0.985 | A |
| 541 | U190 | NC264 | 0.524 | 0.476 | Mixed |
| 542 | U191 | NC290A | 0.01 | 0.99 | A |
| 543 | U192 | NC294 | 0.007 | 0.993 | A |
| 544 | U193 | NC296 | 0.993 | 0.007 | B |
| 545 | U194 | NC296A | 0.992 | 0.008 | B |
| 546 | U195 | NC298 | 0.991 | 0.009 | B |
| 547 | U196 | NC300 | 0.995 | 0.005 | B |
| 548 | U197 | NC302 | 0.988 | 0.012 | B |
| 549 | U198 | NC304 | 0.985 | 0.015 | B |
| 550 | U199 | NC306 | 0.005 | 0.995 | A |
| 551 | U200 | NC310 | 0.004 | 0.996 | A |
| 552 | U201 | NC314 | 0.062 | 0.938 | A |
| 553 | U202 | NC318 | 0.456 | 0.544 | Mixed |
| 554 | U203 | NC320 | 0.528 | 0.472 | Mixed |
| 555 | U204 | NC324 | 0.012 | 0.988 | A |
| 556 | U205 | NC326 | 0.005 | 0.995 | A |
| 557 | U206 | NC328 | 0.003 | 0.997 | A |
| 558 | U207 | NC33 | 0.267 | 0.733 | Mixed |
| 559 | U208 | NC336 | 0.992 | 0.008 | B |
| 560 | U209 | NC338 | 0.991 | 0.009 | B |
| 561 | U210 | NC340 | 0.996 | 0.004 | B |
| 562 | U211 | NC342 | 0.041 | 0.959 | A |
| 563 | U212 | NC344 | 0.026 | 0.974 | A |
| 564 | U213 | NC346 | 0.973 | 0.027 | B |
| 565 | U214 | NC348 | 0.994 | 0.006 | B |
| 566 | U215 | NC350 | 0.962 | 0.038 | B |
| 567 | U216 | NC352 | 0.993 | 0.007 | B |
| 568 | U217 | NC354 | 0.995 | 0.005 | B |
| 569 | U218 | NC356 | 0.772 | 0.228 | Mixed |
| 570 | U219 | NC358 | 0.876 | 0.124 | B |
| 571 | U220 | NC360 | 0.449 | 0.551 | Mixed |
| 572 | U221 | NC362 | 0.414 | 0.586 | Mixed |
| 573 | U222 | NC364 | 0.415 | 0.585 | Mixed |
| 574 | U223 | NC366 | 0.636 | 0.364 | Mixed |
| 575 | U224 | NC368 | 0.033 | 0.967 | A |
| 576 | U225 | ND246 | 0.026 | 0.974 | A |
| 577 | U226 | Oh40B | 0.07 | 0.93 | A |
| 578 | U227 | Oh43 | 0.005 | 0.995 | A |
| 579 | U228 | Oh43E | 0.007 | 0.993 | A |
| 580 | U229 | Oh603 | 0.223 | 0.777 | Mixed |
| 581 | U230 | OH7B | 0.008 | 0.992 | A |
| 582 | U231 | Os420 | 0.017 | 0.983 | A |
| 583 | U232 | P39 | 0.007 | 0.993 | A |
| 584 | U233 | Pa762 | 0.017 | 0.983 | A |
| 585 | U234 | Pa875 | 0.216 | 0.784 | Mixed |
| 586 | U235 | Pa880 | 0.187 | 0.813 | A |
| 587 | U236 | Pa91 | 0.052 | 0.948 | A |
| 588 | U237 | R109B | 0.085 | 0.915 | A |
| 589 | U238 | R168 | 0.062 | 0.938 | A |
| 590 | U239 | R177 | 0.057 | 0.943 | A |
| 591 | U240 | R229 | 0.017 | 0.983 | A |
| 592 | U241 | R4 | 0.018 | 0.982 | A |
| 593 | U242 | SA24 | 0.03 | 0.97 | A |
| 594 | U243 | SC213R | 0.335 | 0.665 | Mixed |
| 595 | U244 | SC357 | 0.208 | 0.792 | Mixed |
| 596 | U245 | SC55 | 0.481 | 0.519 | Mixed |
| 597 | U246 | SD40 | 0.246 | 0.754 | Mixed |
| 598 | U247 | SD44 | 0.141 | 0.859 | A |
| 599 | U248 | Sg1533 | 0.082 | 0.918 | A |
| 600 | U249 | Sg18 | 0.166 | 0.834 | A |
| 601 | U250 | T232 | 0.254 | 0.746 | Mixed |
| 602 | U251 | T234 | 0.304 | 0.696 | Mixed |
| 603 | U252 | T8 | 0.059 | 0.941 | A |
| 604 | U253 | Tx303 | 0.436 | 0.564 | Mixed |
| 605 | U254 | Tx601 | 0.932 | 0.068 | B |
| 606 | U255 | Tzi10 | 0.988 | 0.012 | B |
| 607 | U256 | Tzi11 | 0.758 | 0.242 | Mixed |
| 608 | U257 | Tzi16 | 0.593 | 0.407 | Mixed |
| 609 | U258 | Tzi18 | 0.981 | 0.019 | B |
| 610 | U259 | Tzi25 | 0.576 | 0.424 | Mixed |
| 611 | U260 | Tzi8 | 0.982 | 0.018 | B |
| 612 | U261 | Tzi9 | 0.886 | 0.114 | B |
| 613 | U262 | U267Y | 0.506 | 0.494 | Mixed |
| 614 | U263 | VA102 | 0.04 | 0.96 | A |
| 615 | U264 | Va14 | 0.014 | 0.986 | A |
| 616 | U265 | Va17 | 0.012 | 0.988 | A |
| 617 | U266 | Va22 | 0.1 | 0.9 | A |
| 618 | U267 | Va26 | 0.069 | 0.931 | A |
| 619 | U268 | Va35 | 0.009 | 0.991 | A |
| 620 | U269 | Va59 | 0.008 | 0.992 | A |
| 621 | U270 | Va85 | 0.024 | 0.976 | A |
| 622 | U271 | Va99 | 0.006 | 0.994 | A |
| 623 | U272 | VaW6 | 0.297 | 0.703 | Mixed |
| 624 | U273 | W117Ht | 0.19 | 0.81 | A |
| 625 | U274 | W153R | 0.063 | 0.937 | A |
| 626 | U275 | W182B | 0.01 | 0.99 | A |
| 627 | U276 | W22R | 0.395 | 0.605 | Mixed |
| 628 | U277 | W22 | 0.012 | 0.988 | A |
| 629 | U278 | W64A | 0.01 | 0.99 | A |
| 630 | U279 | WD | 0.048 | 0.952 | A |
| 631 | U280 | Wf9 | 0.005 | 0.995 | A |
| 632 | U281 | Yu796_NS | 0.022 | 0.978 | A |
